# Mechanisms hypothesized for pain-relieving effects of exercise in fibromyalgia: a scoping review

**DOI:** 10.1177/1759720X231182894

**Published:** 2023-07-16

**Authors:** Yuva Venkata Raghava Neelapala, Domenico Mercuri, Luciana Macedo, Steven Hanna, Dylan Kobsar, Lisa Carlesso

**Affiliations:** School of Rehabilitation Science, McMaster University, Hamilton, ON, Canada; School of Rehabilitation Science, McMaster University, Hamilton, ON, Canada; School of Rehabilitation Science, McMaster University, Hamilton, ON, Canada; Department of Health Research Methods, Evidence, and Impact, Faculty of Health Sciences, McMaster University, Hamilton, ON, Canada; Department of Kinesiology, McMaster University, Hamilton, ON, Canada; School of Rehabilitation Science, McMaster University, 1400 Main St. W, IAHS 441, Hamilton, ON L8S 1C7, Canada; Research Institute of St. Joseph’s Hospital, Hamilton, Canada

**Keywords:** fibromyalgia syndrome, mechanisms of action, therapeutic exercise

## Abstract

**Background:**

Exercise is one of the most recommended management strategies by treatment guidelines for fibromyalgia (FM); however, the mechanism through which exercise improves pain in FM is still unknown.

**Objective:**

We aimed to summarize the hypothesized theoretical mechanisms for the pain-relieving effects of exercise in people with FM.

**Eligibility Criteria:**

Randomized controlled trials (RCTs) in English reporting mechanisms for pain-relieving effects of exercise in the ‘Introduction’ and ‘Discussion’ sections and significant within- group or between-group effects of exercise interventions were included.

**Sources of Evidence:**

We searched the databases Ovid MEDLINE(R), EMBASE, CINAHL, COCHRANE, Sports Discuss, and AMED with the keywords: exercise and fibromyalgia until December 2021.

**Charting Methods:**

Two authors independently performed title/abstract, full-text review, and data abstraction using a data abstraction form. The hypothesized mechanisms from individual studies were grouped into three categories.

**Results:**

The literature search resulted in 2147 studies, out of which 220 studies were considered for full-text review. A total of 50 RCTs proposing 29 unique mechanisms for the pain-relieving effects of exercise were included. These mechanisms were divided into three categories: physical, neuro-physiological, and psychological. The neuro-physiological category was further subdivided into exercise-induced hypoalgesia (EIH), pain sensitization, the autonomic system, the immune system, the endocrine system, and miscellaneous categories. The most frequently hypothesized mechanisms were EIH (*n* = 15), autonomic modulation (*n* = 7), improved sleep (*n* = 6), muscle oxygenation (*n* = 6), self-efficacy (*n* = 5), mental health (*n* = 4), and benefits of the aquatic environment (*n* = 12). While all exercise interventions involved FM patients, most of the supporting evidence for these mechanisms was cited from previous studies conducted on healthy samples. No studies performed analyses to demonstrate causal associations between the mechanisms and outcomes.

**Conclusion:**

Multiple mechanisms were hypothesized for the positive influence of exercise in people with FM. Future studies using causal analyses, such as mediation analysis, are recommended to validate these mechanisms.

## Background

Fibromyalgia (FM) is defined by the presence of widespread pain and affects approximately 2% of the general population^1^ with an estimated prevalence of 1.4% among Canadian individuals aged 12 and above.^2^ FM poses a significant health burden negatively affecting physical and mental health, and quality of life.^3^ Additionally, FM is associated with significant direct and indirect health care costs with a recent study in Canada estimating annual medical expenditures of CAD 3800 per person.^4^ The majority of these costs are due to the purchase of over-the-counter pain medications indicating a significant pain burden, which also results in 3 weeks of lost work per year approximately.^4^ A multidisciplinary treatment approach with an emphasis on non-pharmacological treatments has been suggested to improve pain, fatigue, and physical function in the management of FM.^5^ However, most commonly prescribed treatments for FM report only modest effectiveness, and satisfactory or clinically important symptom reduction is not attained in most individuals.^5,6^

Canadian and European Treatment guidelines recommend exercise as one of the major components of the first-line treatment for FM.^5,6^ This recommendation is substantiated by a large number of systematic reviews suggesting the effectiveness of various types of exercise interventions to reduce symptoms in FM.^7^ The most significant positive effects of exercise in FM are identified on the outcomes of pain intensity and quality of life.^7^

Although research on the effectiveness of exercise in FM has been increasing in volume, the underlying mechanisms that explain the beneficial effects of exercise in FM have not been well studied. For example, exercise may counteract the physical deconditioning, or the abnormal pain processing associated with FM; however, these mechanisms have not been reviewed previously. Due to perceived and experienced pain exacerbation during exercise, participation in exercise programs remains a challenge for some individuals with FM.^8^ Better knowledge of the mechanisms of action underlying exercise may translate into improved prescription and increased exercise adherence through enhanced education and self-management strategies. More specifically, understanding the mechanisms of exercise may help to guide the development of education strategies or tailoring exercise regimes according to a patient’s presentation. Lastly, understanding the mechanism of action can guide further research on moderators and mediators of treatment outcomes after exercise interventions in FM.

Given no previous reviews have summarized the mechanisms for the pain-relieving effects of exercise in FM, our scoping review aims to summarize the hypothesized theoretical mechanisms for the pain-relieving effects of exercise in randomized controlled trials (RCTs) on exercise in FM. A secondary objective was to identify any analyses involving hypothesized mechanisms and pain outcomes. A scoping review methodology was used for this study as the research question attempts to identify the concepts and theories on exercise mechanisms in FM.

## Methods

This scoping review follows the methodology initially proposed by Arksey and O Malley and later expanded by Levac *et al.*^9^ which includes (1) Identifying the research question; (2) identifying relevant studies; (3) selecting studies; (4) charting, collating, and summarizing the data; (5) reporting the results. The sixth step of consultation was not conducted in the review. The results are reported as per Preferred Reporting Items for Systematic Reviews and Meta-Analyses (PRISMA) extension guidelines for scoping reviews (2020).^10^

**Step One: Identifying the research questions:** This scoping review answers the following research question:

What are the hypothesized theoretical mechanisms explaining exercise-related improvements in pain in people with FM?

What are the analyses used in studies evaluating the relationship (causal or not) between the mechanisms and pain outcomes?

**Step Two: Identifying relevant studies:** A systematic search was conducted in the databases Ovid MEDLINE(R), EMBASE, CINAHL, COCHRANE, Sports Discuss, and AMED from inception to December 2021. The search strategy was developed in consultation with a University Health Sciences librarian with the primary keywords; exercise and FM combined with Boolean operators. The search strategy used the limits for RCTs, English language, and humans (Supplemental Appendix 1).

Inclusion criteria:

(i) RCTs published in the English language that investigated the effects of exercise intervention and reported mechanisms for the pain-relieving effects of exercise were included. The exercise interventions considered were aerobic, muscle strengthening, flexibility, aquatic, yoga, Tai-Chi, and Qigong as these are the commonly investigated exercise interventions in FM.
(ii) The studies must include participants with FM based on a recommended criteria or diagnosed by a physician.
(iii) Studies must report statistically significant within or between-group differences of the exercise intervention on pain intensity outcomes, that is, pain intensity (e.g. visual analogue scale, numeric pain rating scale), pain thresholds (e.g. pressure pain thresholds), and myalgic pain score (tenderness at 18 tender points evaluated on 0–4 scale). The number of tender points was not considered eligible. If within-group differences were not reported in the studies, the statistical significance was ascertained by calculating mean differences with 95% confidence intervals from the mean and standard deviation values for pain outcomes.
(iv) Studies must report the mechanisms for pain-relieving effects of exercise in the Introduction, Methods, or Discussion section of the article.

Exclusion criteria:

(i) Studies with FM participants along with any other conditions or comorbidities.
(ii) Studies were excluded if the sample size is <10 per group or if the mean differences for pain outcomes are not statistically significant.
(iii) Protocols of RCTs and non-randomized studies were excluded.
(iv) Studies were excluded if the exercise intervention was combined with another type of intervention or if the mechanisms were not reported.
**Step three: Study selection.** Two reviewers (YV and DM) performed the study selection independently at all stages (title abstract, and full-text review) through Covidence systematic review software, and disagreements were resolved by a third reviewer (LC). Pilot testing of 10 studies was conducted to standardize the decision-making and agreement for study selection. In the first stage, the titles and abstracts of the retrieved studies were screened for the eligibility criteria independently by the two reviewers (YV and DM) to be considered for full-text review. In the second stage, full-text articles were reviewed independently by the same two reviewers (YV and DM) to determine eligibility for final inclusion in the review. Reference lists of the retrieved articles were also searched.
**Step four: Charting the data:** A standardized data extraction form was created by the review team and initially piloted on five articles. Any issues that resulted in the pilot testing were clarified through consultation. The following items were extracted from the studies by the two reviewers (YV and DM) independently.
 • Publication Summary: Authors, Year
 • Population Characteristics: Age, sex, race, body mass index (if reported)
 • Intervention Details: Frequency, Type, Intensity, Duration, and the details of the comparator intervention.
 • Sample size and the mean differences or effect size for the intervention
 • Reported Mechanisms: Hypothesized mechanisms for exercise-related improvements in pain in people with FM reported within the included studies.
 • Pain intensity outcomes reported in the included studies.
 • Any exclusion criteria that may influence the pain outcome in either direction (increase or decrease).
 • Relevant analyses reported in the studies in support of the hypothesized mechanisms.
**Step five: Collating, summarizing, and reporting the results:** During data extraction, the ‘Introduction’, ‘Methods’, and ‘Discussion’ sections of the included studies were systematically screened for the underlying mechanisms in addition to the results. In the next step, the underlying mechanisms listed in the studies were grouped into common concepts. Finally, the concepts were placed in relevant ordered categories corresponding to scientific foundations explaining the mechanisms of exercise in FM. Also, the analyses including the relationship between pain outcomes and the hypothesized mechanisms were summarized.

## Results

**Figure 1.**
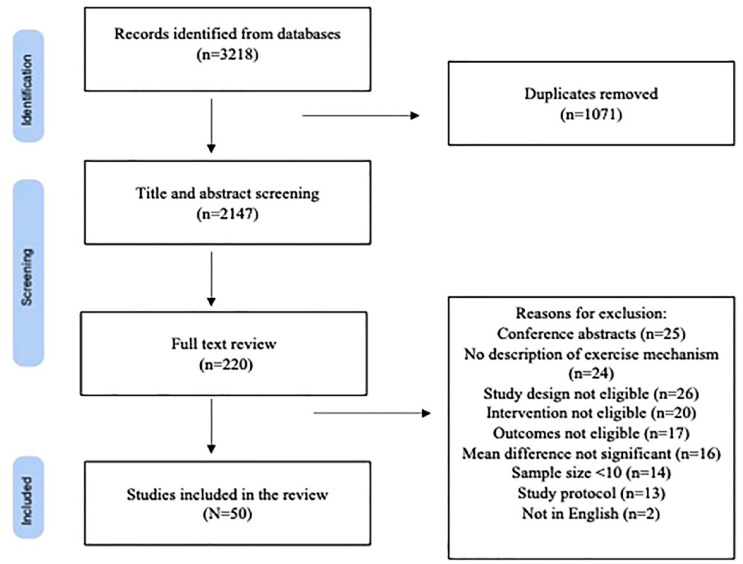
PRISMA flow diagram.

The initial search resulted in 2147 studies after the removal of duplicates for the title and abstract screening, out of which 220 studies were considered for full-text review. In total, 50 RCTs were considered eligible for inclusion in the review. Figure 1 shows the PRISMA flow diagram along with reasons for exclusion, with common reasons being no description of exercise mechanisms, non-randomized trials, pain outcomes not reported, and mean differences not being significant. The type of exercise interventions investigated in the RCTs are as follows: aquatic exercise (17 trials),^11–27^ aerobic exercise (12 trials),^14,25,28–39^ resistance exercise (13 trials),^15,21,31,35,40–48^ flexibility exercise (7 trials),^28,32,40,41,43,45,49^ Qigong (2 trials),^50,51^ Tai-Chi (2 trials),^52,53^ yoga (1 trial),^54^ high-intensity interval training (1 trial),^55^ and mixed exercise intervention (14 trials).^11,17,19,25,26,30,36,54,56–60^Table 1 shows the complete list of mechanisms with the number of studies endorsing a specific mechanism.

**Table 1.** Summary of mechanisms proposed in the studies (number of studies endorsing the mechanism).

Physical (*n* = 45)	Neuro-physiological (*n* = 48)	Psychosocial (*n* = 18)
Reduces Gravitational forces (11) Muscle tension (2) Deconditioning (2) Sensitivity of tender points (1) Inflammation (1) Stimulates Tactile, Mechano & Thermo receptors (3) Improves Removal of chemical mediators Sleep (6) Relaxation (5) Muscle oxygenation (6) Flexibility (2) Joint mobility (1) Body mechanics (1) Balance (1) Neuromuscular control (1) Peripheral adaptations (1) (Increase of oxidative enzymes, capillary density, high energy phosphate) Maintains Healthy body composition (1)	Exercise-induced hypoalgesia (15) Mediated by Opioid (e.g. increased endorphins) and non-opioid mechanisms. (e.g. growth hormone and corticotrophin) Autonomic system related (7) Reduces sympathetic hyperactivity, improves autonomic control and sympathovagal balance. Immune system related (2) Improves immune regulation (C-reactive protein, IL-6) (1) Down regulates production of pro-inflammatory cytokines (1) Endocrine system related. Ameliorates hypothalamic-pituitary axis dysfunction (4) Neurotransmitters: Increases the release of serotonin (2), angiotensin II, nitric oxide, cytokines, (1) Improves the concentration of dopamine (1), serum cortisol, Adrenocorticotropic Hormone H, Growth Hormone, Insulin-like Growth Factor-1 (1), nerve growth factor and brain-derived nerve growth factor (1) Pain modulation (6) Modulates cortical activity (2) through long-term potentiation (1) Reduces peripheral and central sensitization (1) Promotes neuroplasticity of Central Nervous System (1) Inhibition of pain in Central Nervous System (1) Miscellaneous (2) Improves flow of Qi (vital energy), body awareness	Improves Self-efficacy (5) Mental health (4) Pain acceptance (1) Belonging to Group (1) Reduces Kinesiophobia (2) Psychological distress (1) Catastrophizing (1) Promotes Placebo effects (1) (supervision) Euphoria (1) Distraction from pain (1)

Overall, 29 unique mechanisms for the pain-relieving effects of exercise were hypothesized by the authors in the included RCTs. These hypothesized mechanisms were grouped into three primary categories: physical (e.g. improving body mechanics), neuro-physiological [e.g. exercise-induced hypoalgesia (EIH)], and psychological (e.g. reducing stress). The neuro-physiological category of mechanisms was further categorized into EIH, pain modulation, the autonomic system, the immune system, the endocrine system, and miscellaneous categories. However, these categories may not be considered distinct and overlapping effects could be possible between these mechanisms. The physical (*n* = 45 times) and neurophysiological (*n* = 48 times) mechanisms were described more often than the psychological (*n* = 18 times) categories and the authors hypothesized more than one mechanism frequently. The examples of verbatim text described in the studies are reported below.

### Physical category

In the physical category, the mechanisms of pain relief due to the aquatic environment (i.e. buoyancy, thermal effects, stimulation of tactile, mechanoreceptors, and relaxation) were more frequently identified (*n* = 12),^11,13,15,16,18–25^ followed by improvements in sleep (*n* = 6),^30,32,38,40,52,59^ muscle oxygenation (*n* = 7),^12–14,30,31,40,58^ and relaxation (*n* = 7).^11,14,15,20,23,52,54^

#### Example of text verbatim

The pool-based environment, with its characteristics of buoyancy (which reduces the effects of gravity), the resistance of water (which reduces the impact on joints, muscles, and tendons), and the relaxing effect water has on muscles, prevent possible post-exercise pain and foster the benefits produced by the training session.^11^

### Neurophysiological

EIH (*n* = 15)^12,13,17,22,23,26,28–30,32–34,37,58,59^ was the most frequent mechanism described in the neurophysiological category with autonomic (*n* = 7)^22,38,43,50,53,54,59^ and pain modulation (*n* = 5)^21,31,33,43,60^ mechanisms being hypothesized in approximately an equal number of studies.

#### Examples of text verbatim

a. Exercise-induced hypoalgesia

 It is a widely accepted hypothesis that activation of the endogenous opioid system during exercise plays a key role in the analgesic response mechanism.^40^

b. Pain sensitization

 Strength or aerobic exercise could enhance muscle oxygenation, which could lead to reductions in peripheral and central sensitization and improvements in clinical pain.^31^

c. Autonomic system

 It is already known that Fibromyalgia syndrome (FMS) is associated with a sympathetic hyperactivity that may be related to its pathogenesis, and yoga as well as meditation may play an important role in reducing this hyperactivity.^54^

d. Immune system

 A possible underlying mechanism that mediated the symptom improvements observed in the experimental group might be the immune state regulation through Qigong including C-reactive protein, interleukin-6.^50^

e. Endocrine system

 The improvements in the symptoms of FMS after an exercise program might be partially attributed to amelioration of disorder in the hypothalamic-pituitary-adrenal (HPA) axis.^28^

f. Miscellaneous

 Pain-relieving effects of Qigong improving the flow of vital energy.^51^

### Psychosocial

In the psychosocial category, improvements in pain were more frequently hypothesized due to improved self-efficacy (*n* = 4)^19,35,45,56^ and mental health (*n* = 4).^32,40,52,56^

#### Examples of text verbatim

Beneficial effects of the intervention ‘per se’ due to the belonging or a therapeutic group or beneficial effects on psychological distress and mental health could be involved.^35^

The authors of most studies hypothesized mechanisms for exercise interventions in general (*n* = 29) while others have reported mechanisms for the specific exercise interventions (e.g. Aquatic exercise (*n* = 12)^11,13,15,16,18–25^ Aerobic exercise (*n* = 4),^33,34,37,38^ Resistance exercise (*n* = 4),^42–44,48^ Flexibility exercise (*n* = 2),^41,49^ Qigong (*n* = 2),^50,51^ Yoga (*n* = 1),^54^ and Tai Chi (*n* = 2)^52,53^). Aerobic exercise has been proposed to stimulate EIH, decrease sympathetic activity, improve psychological status, and promote long-term potentiation in the central nervous system.^33,34,37,38^ Resistance exercise specifically has been proposed to reduce pain by reversing deconditioning,^42^ promoting autonomic modulation which improves the production of neurotransmitters.^43,44^ Flexibility exercise has been proposed to reduce the sensitivity of tender points, improve freedom of movement, and reduce kinesiophobia and thus reducing pain.^41,49^ Most of the pain-relieving effects of aquatic exercise are attributed to the physical properties of water (e.g. buoyancy and reduced gravitational forces), tactile stimulation, and effects of the temperature of water.^11,13,15,16,18–25^ Tai-Chi^52,53^ has been proposed to reduce pain through autonomic modulation, relaxation, improved sleep, and vital energy whereas Qigong has been proposed to have similar effects in addition to modulation of hypothalamic-pituitary axis dysfunction and the reduction of hormonal stress levels.^50,51^ Lastly, Yoga has been proposed to reduce pain by reduced muscle tension and sympathetic hyperactivity.^54^ While all included studies conducted research on FM patients, the majority of studies cited research conducted on healthy individuals in support of the hypothesized mechanism.

#### Additional analyses reported in the included studies

Six^13,14,38,44,53,56^ out of the 50 RCTs studies examined the relationship between changes in the outcomes of a hypothesized mechanism and the corresponding improvements in pain. Table 2 describes the studies reporting a correlation/regression analysis between outcomes of hypothesized mechanisms and pain post-intervention. In one study, a significant correlation (*r* = 0.68) was identified between changes in autonomic modulation (i.e. heart rate variability) and pain intensity following Tai-Chi intervention.^53^ The remaining five studies reported no significant relationship between the outcomes of the hypothesized mechanisms (e.g. improved aerobic fitness) and changes in pain outcome. Supplemental Appendix 2 summarizes the data abstraction items, namely baseline characteristics of the participants, details of the intervention and comparator, pain outcomes reported.

**Table 2.** Correlation/regression analyses reported between proposed outcomes and pain.

Author	Exercise intervention	Outcomes of mechanisms	Pain outcomes	Analyses reported and results
Andrade *et al*. (2019)	Aquatic aerobic exercise	Cardiorespiratory fitness measured using Vo2 max	PPT, VAS	*Correlation*: No significant correlation was identified between improvements in Vo2max and pain outcomes
Assis *et al*. (2006)	Aquatic Aerobic exercise	Aerobic gain measured by peak oxygen uptake	VAS	*Correlation*: No correlation was observed between aerobic gain and VAS
Valim *et al*. (2003)	Aerobic exercise	Cardiorespiratory fitness measured by peak oxygen uptake at ventilatory anaerobic threshold	VAS	*Multiple linear regression*: No relationship between improvements in VAT and VAS in the aerobic exercise group (*p* value = 1.00)
Wong *et al*. (2018)	Tai Chi	Cardiac autonomic modulation measured by heart rate variability	VAS	*Correlation*: Significant positive relationship was identified between changes in Heart Rate Variability and pain in the Tai Chi group (*r* value = 0.68; *p* value <0.05)
Jablochkova *et al*. (2019)	Resistance exercise	Markers of pain sensitization: Nerve growth factor and brain derived neurotropic factor	PPT, VAS	*Multiple linear regression*: No significant relationship between changes in Nerve Growth Factor/Brain Derived Neurotrophic Factor and changes in pain
Garcia-Martinez *et al*. (2012)	Combined exercise	Self-esteem measured using Rosenberg self-esteem scale	Bodily pain measured using SF-36	*Correlation*: No significant relationship Improvements in self-esteem and improvements in pain (*r* value = 0.37; *p* value >0.05)

PPT, pressure pain threshold; VAS, visual analogue scale.

## Discussion

Our scoping review identified 29 unique mechanisms hypothesized for the pain-relieving effects of exercise in RCTs that examined the effectiveness of exercise interventions on pain outcomes in FM. We summarized these various mechanisms hypothesized by the authors under three potentially inter-related categories (physical, neurophysiological, and psychological). Overall, the most commonly hypothesized mechanisms were EIH, benefits of aquatic environment, autonomic and pain modulation, improved sleep, self-efficacy, mental health, and tissue oxygenation.

Most studies (except for aquatic exercise) have proposed mechanisms for exercise in general, with a smaller number of studies proposing mechanisms specific to the exercise intervention. Twelve out of 17 studies proposed mechanisms specific to aquatic exercise.^11,13,15,16,18–25^ which include reduced gravitational forces, and relaxation. Mechanisms specific to aerobic and resistance exercise were proposed in four out of 12 and 13 trials respectively. More commonly, aerobic exercise has been proposed to reduce pain through EIH^33,34^ whereas resistance exercise was proposed to reverse deconditioning.^42,43^ Two out of seven trials proposed mechanisms specific to flexibility exercise which included reducing the sensitivity of tender points.^41,49^ All the studies on Tai-Chi, Qigong, and Yoga proposed mechanisms specific to these interventions which most commonly included autonomic modulation.^50–54^

Though exercise is strongly recommended by international guidelines in the management of FM, the potential treatment targets of exercise interventions (e.g. to improve aerobic fitness) in this population are unknown. However, due to the complex disease mechanisms underlying the clinical presentation of FM, the identification of targets for exercise interventions is complicated. A possible reason for the limited understanding of the treatment targets for exercise intervention could be the uncertainty around the mechanisms through which exercise results in positive effects. In other musculoskeletal disorders such as low back pain (LBP) and knee osteoarthritis (OA), exercise interventions are usually targeted to improve neuromuscular impairments such as motor control, muscle strength, and flexibility.^61–63^ We identified that, exercise has been proposed to counteract the underlying disease mechanisms in people with FM which include central sensitization, dysfunction in the autonomic system, and hypothalamic-pituitary axis.^64^ This provides mechanistic insights for improved pain outcomes in the studies included in our review. However, it should be noted that none of the RCTs performed analyses or cited research that demonstrated the causality of the mechanisms.

## Physical mechanisms

A higher number of studies (*n* = 17)^11–27^ that delivered aquatic exercise were included in our review compared to the other types of exercise interventions. It has been proposed that the pain-relieving effects specific to aquatic exercise are mostly due to the physical properties of water (e.g. reduced gravitational forces due to buoyancy) and exercising in the warm aquatic environment (thermal and tactile stimulation).^11,13,15,16,18–20^

Several studies (*n* = 6)^30,32,38,40,52,59^ hypothesized that the pain-relieving effects of exercise, in general, may be due to improved sleep. Sleep disturbances are a cardinal feature of FM with poor sleep having a bidirectional relationship with pain intensity^65^ and exercise interventions result in small improvements in sleep quality in people with FM.^66^ The mechanisms for improved sleep and pain with exercise are overlapping such as reduced sympathetic hyperactivity and hypothalamic-pituitary axis dysfunction.^66^ However, no studies in our review have cited research that showed a causal relationship between improvements in sleep resulting in improvements in pain due to exercise and therefore, the plausible mediating role of sleep quality may be a potential area to investigate further.

Few studies (*n* = 3)^13,14,55^ included in our review hypothesized that improved physical conditioning (indicated by increased aerobic fitness, muscle strength, and improved body composition) may be the mechanism for pain reduction after exercise intervention. These studies have cited cross-sectional research (with no exercise intervention) demonstrating an association between cardiorespiratory fitness, body composition and pain sensitivity, and symptom severity, respectively.^67,68^ In FM, aerobic and mixed exercise has been found to improve aerobic fitness (measured using VO2 max), and muscle strength of knee extensors and flexors significantly.^13,14,27,38^ However, improvements in pain were not correlated with exercise-related improvements in either aerobic fitness or muscle strength, based on the correlation/regression analyses reported in these studies included in our review.^13,14,27,38^ Therefore, it is still unclear whether the improvements in pain are due to increased aerobic fitness or muscle strength and further research is required to determine whether aerobic fitness or muscle strength are potential targets of exercise in FM.

Another potential mechanism hypothesized is the increase in muscle oxygenation due to exercise.^13,31,58^ Pain in FM is attributed to reduced muscle oxygenation due to impaired capillary circulation and the associated peripheral and central sensitization.^69^ A case-control study showing improved oxygen kinetics in muscles after 8 weeks of aerobic exercise supports this mechanism.^70^ It has been proposed that improved microcirculation after exercise may reduce peripheral and central sensitization and thus reduce pain in individuals with FM.^31^ Similar to these earlier studies, a recent systematic review reported positive effects of hyperbaric oxygen therapy on pain outcomes in FM individuals. The proposed mechanisms for such benefits include improved oxygenation to the ischemic tissues, reduced activation of glial cells and other inflammatory mediators causing an anti-inflammatory effect, and improved metabolic function.^71^ Lastly, improved flexibility specifically after stretching exercise was reported as a mechanism for pain relief,^41,49^ but no studies investigating the relationship between improvements in flexibility and pain were cited. It has been proposed that stretching exercise reduces the sensitivity of tender points and fear of movement associated with pain.^41^

## Neurophysiological mechanisms

EIH, which is defined as ‘reduction in pain following an exercise intervention’ is a commonly cited mechanism. Numerous underlying mechanisms have been proposed to explain the phenomenon of EIH, of which descending modulation of pain by activation of opioid and cannabinoid systems is most commonly reported.^72^ The muscle contractions during exercise have been proposed to stimulate the central descending pain pathways and increase the release of endogenous opioids which produces the analgesic effect.^72^ Despite EIH being the most frequently cited mechanism for pain-relieving effects of exercise, most of the studies have cited research conducted on healthy individuals.^12,13,17,22,23,26,28–30,32–34,37,58,59^ This is important to note when other studies (not included in our review) have identified that individuals with FM have impaired EIH and increased pain sensitivity following various types of exercise.^73–75^ A recent systematic review that included studies on individuals with FM concluded that there is insufficient evidence to determine whether EIH is impaired or functional in chronic musculoskeletal pain conditions.^76^ Therefore, further work is required to implicate EIH as a plausible underlying mechanism for the pain-relieving effects of exercise in FM.

Another common mechanism hypothesized in this category is the modulatory effect of exercise on the autonomic system, by reducing the hyperactivity of the sympathetic nervous system and the accompanying stress response.^77^ Compared to healthy controls, individuals with FM have been found to have autonomic dysfunction which is implicated as an important cause for symptoms.^78^ A recent systematic review concluded that there is preliminary evidence in support of aerobic exercise reducing autonomic dysfunction in FM, but not for resistance exercise and more research is required to arrive at definitive results.^77^ Studies included in our review hypothesized improved autonomic function (reduction in sympathetic hyperactivity) as a potential mechanism for pain reduction following aerobic, resistance, Tai-Chi, yoga, and Qigong interventions.^22,38,43,50,53,54,59^ However, only one study included in our review using a Tai Chi intervention reported that the changes in outcomes of autonomic dysfunction were correlated with changes in pain levels.^53^ Tai Chi has been proposed to reduce pain by improving sympathovagal balance (reduction in sympathetic activity and enhancement and parasympathetic activity).^53^ No other studies investigated the causal relationship between autonomic function and pain outcomes following exercise intervention warranting further research in this area.

Exercise has been proposed to reduce pain by reversing the HPA axis dysfunction through its effects on inflammatory, and neuroendocrine markers and other neurotransmitters associated with immune and endocrine functions.^28,29,50,58^ The pathophysiology of FM includes HPA dysfunction and chronic low-grade inflammation characterized by an increase in serum pro-inflammatory markers.^78^ Preliminary evidence was cited in the included studies, that showed exercise (both single session and long term) increased growth hormone and serum cortisol levels (markers of HPA function) in individuals with FM.^79–81^ On the other hand, a systematic review reported that the effects of single-session exercise (25–45 min) on pro- and anti-inflammatory biomarkers are unclear in FM; however, an overall anti-inflammatory effect of exercise was observed.^82^ In line with these results, one study in our review reported that resistance exercise did not significantly change the neurotrophins (plasma nerve growth factor and brain-derived neurotrophic factor) nor did they have a significant relationship with pain outcomes.^44^

Few studies identified in our review have proposed that exercise reduces pain through the reduction of peripheral and central sensitization.^21,31,33,43,60^ Peripheral factors such as anatomical changes in muscle fibers, and abnormalities in microcirculation cause peripheral sensitization, which in turn drive central sensitization in FM.^31,83^ Exercise has been proposed to counteract peripheral and central sensitization by improving peripheral oxygenation, increasing serotonin levels, and stimulating pain inhibitory process in the rostro ventral medulla.^31,84^ Recent systematic reviews conclude that exercise reduces pain sensitivity (a marker of pain modulation) in chronic musculoskeletal pain conditions including FM^85,86^ indicating pain modulation as a potential mechanism for pain relief. In summary, though there is preliminary evidence in support of the neurophysiological mechanisms for pain reduction after exercise, the mediating role of these mechanisms needs further validation.

## Psychosocial mechanisms

Studies identified in our review have hypothesized that exercise reduces pain by improving self-efficacy, providing social interaction during the exercise session, and reducing the impact of psychological factors (e.g. kinesiophobia, and catastrophizing) associated with pain.^19,35,42,49^ Pain relief through improved self-efficacy^19,35,45,56^ and mental health^32,49,52,56^ were the most frequently proposed mechanisms in this category. Regular exercise has been proposed to increase confidence to perform various physical tasks and reduces accompanying pain with these activities.^8^ Studies have cited Buckelew *et al.* and colleagues who were among the first to report the association between self-efficacy, exercise, and pain in FM.^87,88^ The changes in self-efficacy and not group allocation predicted changes in pain outcomes following rehabilitation interventions (biofeedback relaxation, exercise, combined biofeedback and exercise, education).^88^ One study in our review reported that 12 weeks of a combined exercise program led to significant improvements in self-esteem, and quality of life (SF-36), and the increased self-esteem scores were positively correlated with the mental health component of the Short Form-36.^56^ However, similar relationship was not observed between self-esteem scores and pain scores.^56^ Another study showed that 8 weeks of low-impact combined exercise led to positive effects on several psychological variables (pain catastrophizing, pain acceptance, anxiety, and stress).^57^ The authors of this study hypothesized that exercise promotes distraction, reduces rumination associated with pain and thus reduces pain.^57^

Another frequently reported mechanism was the positive effects of exercise on mental health and psychological distress^49,52^; however, studies included have not explained the causal relationship between these factors (i.e. mental health and psychological distress), exercise, and pain. Previous research has shown that anxiety and depression are very common in FM^89^ and exercise has been proposed to positively affect mental health and pain by improving the neurotransmitter release in the brain.^90^ Lastly, Larsson *et al.*,^47^ demonstrated that 15 weeks of resistance exercise resulted in improved pain acceptance, and reduced fear avoidance, and identified a significant positive relationship between improvements in pain scores and global impression of changes scores. This might imply that the overall perceived benefit from the intervention may also be a reason for the pain-relieving effects of exercise in FM. Overall, exercise appears to have positive effects on various psychological factors associated with pain in FM and the mediating role of these mechanisms on pain severity might benefit further investigation.

The results of our review are comparable to similar reviews in the areas of knee OA, and chronic LBP.^61,62^ Beckwee *et al.*,^62^ reported six categories of potential mechanisms for the positive effects of exercise in knee OA. Wun *et al.*^61^ identified 33 proposed mechanisms of action for exercise in chronic LBP and grouped these mechanisms under five themes. These authors identified that the neuromuscular theme has been proposed most frequently, while our review identified that the neurophysiological category was the most frequently proposed mechanism for exercise in general. This may reflect that in people with FM, a more general exercise approach has been studied in contrast to those with Chronic Low Back Pain where a specific exercise approach targeting the neuromuscular impairments has been delivered. In addition, it is not yet clear whether the hypothesized mechanisms could be attributed to a specific exercise or whether these mechanisms might be working across all types of exercise. Knowing the specificity of exercise effects may guide decision-making on the selection of the type of exercise intervention tailored to the underlying pathophysiology of the disease. However, it must be noted that exercise may reduce pain through multiple interconnected pathways, and knowing the most relevant working mechanism may possibly enhance the effectiveness of exercise.

## Limitations

We used a comprehensive search strategy to identify RCTs reporting the most commonly investigated exercise interventions for FM and their hypothesized mechanisms; however, there may be observational study designs or secondary analyses of the RCT data that we were unable to capture in our search. In 6 of the 50 studies included, we identified the reported associations between the outcomes of hypothesized mechanism and pain. In addition, we limited the eligibility criteria to RCTs with the positive effects of exercise on pain outcomes. The inclusion of studies with non-significant effects may have provided alternative hypotheses for the mechanisms identified. However, our review provided an initial comprehensive summary of the hypothesized mechanisms for pain-relieving effects of exercise in FM and provides several potential directions for future research, thus achieving the recommended goals of conducting a scoping review.

## Implications for future research and clinical practice

Based on the studies identified in the review, we propose that there is a great need for further research investigating the mechanisms underlying pain-relieving effects of exercise in FM as most of the articles cited research conducted on healthy individuals. Therefore, first, it may be viable to conduct a systematic review including studies of all designs investigating the relationship between the outcomes of the hypothesized mechanisms and pain outcomes. In addition, there has been growing interest recently to understand and evaluate mechanisms through which various treatments work for chronic musculoskeletal pain conditions.^91–93^ Therefore, future RCTs investigating the effectiveness of exercise in FM may consider developing exercise interventions to target specific mechanisms underlying pain modulation and identify potential moderators and mediators of the exercise intervention. For example, one trial identified a significant correlation between heart rate variability and pain outcomes after Tai Chi intervention, which may indicate that improving autonomic function could be a treatment target for the exercise intervention. Our review summarized several modifiable mechanisms plausibly mediating the pain-relieving effects of exercise which need further investigating by analyses establishing causation (e.g. mediation). Such trials facilitate the development of targeted exercise interventions with specific goals and objectives and can optimize exercise prescription with specific dosages for FM in clinical practice. The knowledge of mechanisms presented in our review may help to inform the development of education strategies to increase the uptake of exercise intervention in FM.

## Conclusion

In conclusion, this scoping review summarized the hypothesized mechanisms for the pain-relieving effects of exercise for people with FM. Though multiple mechanisms were hypothesized for the pain-relieving effects of exercise, most of the studies included in our review cited research conducted on normal healthy individuals in support of the mechanisms. Therefore, it is not possible to imply that similar mechanisms are operational in individuals with FM and there is a strong need for research investigating the underlying mechanisms for pain-relieving effects of exercise in FM. This review will help to guide further research optimizing exercise strategies for people with FM by identifying the possible mechanisms of action through mediation or moderator analysis.

## Supplemental Material

sj-docx-1-tab-10.1177_1759720X231182894 – Supplemental material for Mechanisms hypothesized for pain-relieving effects of exercise in fibromyalgia: a scoping review Click here for additional data file. Supplemental material, sj-docx-1-tab-10.1177_1759720X231182894 for Mechanisms hypothesized for pain-relieving effects of exercise in fibromyalgia: a scoping review by Yuva Venkata Raghava Neelapala, Domenico Mercuri, Luciana Macedo, Steven Hanna, Dylan Kobsar and Lisa Carlesso in Therapeutic Advances in Musculoskeletal Disease

## Declarations

**Ethics approval and consent to participate:** Not applicable.

**Consent for publication:** Not applicable.

**Availability of data materials:** Not applicable.
