# Supplementary material for: Mechanisms hypothesized for pain-relieving effects of exercise in fibromyalgia: a scoping review
Source: Ther Adv Musculoskelet Dis. 2023 Jul 16;15:1759720X231182894. doi: 10.1177/1759720X231182894 (PMC10356998; doi:10.1177/1759720X231182894)
Supplement: sj-docx-1-tab-10.1177_1759720X231182894 – Supplemental material for Mechanisms hypothesized for pain-relieving effects of exercise in fibromyalgia: a scoping review [file sj-docx-1-tab-10.1177_1759720X231182894.docx]

**Appendix 1: Search Strategy**

**Embase <1974 to 2021 December 15>**

1 exp fibromyalgia/

2 fibromyalgia.mp.

3 fibromyalgia syndrome.mp.

4 fibrositis.mp.

5 exp exercise/

6 exercise.mp.

7 exercise therap*.mp.

8 therapeutic exercise.mp.

9 exp aerobic exercise/

10 aerobic training.mp.

11 aerobic exercise.mp.

12 exp resistance training/

13 resistance training.mp.

14 resistance exercise.mp.

15 strength training.mp.

16 exp weight lifting/

17 weight lifting.mp.

18 exp muscle training/

19 muscle training.mp.

20 muscle strengthening.mp.

21 strengthening exercise.mp.

22 exp hydrotherapy/

23 hydrotherap*.mp.

24 aquatic therap*.mp.

25 exp aquatic exercise/

26 aquatic exercise.mp.

27 underwater exercise*.mp.

28 stretching exercise*.mp.

29 exp muscle stretching/

30 muscle stretching.mp.

31 exp stretching exercise/

32 flexibility training.mp.

33 exp yoga/

34 yoga.mp.

35 yoga therap*.mp.

36 therapeutic yoga.mp.

37 yoga pose*.mp. 94

38 yoga posture*.mp.

39 yoga asana*.mp.

40 exp Tai Chi/

41 Tai Chi.mp.

42 exp qigong/

43 qigong.mp.

44 exp physiotherapy/

45 physiotherap*.mp.

46 physical therap*.mp.

47 exp rehabilitation/

48 rehabilitation.mp.

49 1 or 2 or 3 or 4

50 5 or 6 or 7 or 8 or 9 or 10 or 11 or 12 or 13 or 14 or 15 or 16 or 17 or 18 or 19 or 20 or 21 or 22 or 23 or 24 or 25 or 26 or 27 or 28 or 29 or 30 or 31 or 32 or 33 or 34 or 35 or 36 or 37 or 38 or 39 or 40 or 41 or 42 or 43 or 44 or 45 or 46 or 47 or 48

51 49 and 50

52 (Randomized controlled trial/ or Controlled clinical study/ or random$.ti,ab. or randomization/ or intermethod comparison/ or placebo.ti,ab. or (compare or compared or comparison).ti. or ((evaluated or evaluate or evaluating or assessed or assess) and (compare or compared or comparing or comparison)).ab. or (open adj label).ti,ab. or ((double or single or doubly or singly) adj (blind or blinded or blindly)).ti,ab. or double blind procedure/ or parallel group$1.ti,ab. or (crossover or cross over).ti,ab. or ((assign$ or match or matched or allocation) adj5 (alternate or group$1 or intervention$1 or patient$1 or subject$1 or participant$1)).ti,ab. or (assigned or allocated).ti,ab. or (controlled adj7 (study or design or trial)).ti,ab. or (volunteer or volunteers).ti,ab. or human experiment/ or trial.ti.) not (((random$ adj sampl$ adj7 ("cross section$" or questionnaire$1 or survey$ or database$1)).ti,ab. not (comparative study/ or controlled study/ or randomi?ed controlled.ti,ab. or randomly assigned.ti,ab.)) or (Cross-sectional study/ not (randomized controlled trial/ or controlled clinical study/ or controlled study/ or randomi?ed controlled.ti,ab. or control group$1.ti,ab.)) or (((case adj control$) and random$) not randomi?ed controlled).ti,ab. or (Systematic review not (trial or study)).ti. or (nonrandom$ not random$).ti,ab. or "Random field$".ti,ab. or (random cluster adj3 sampl$).ti,ab. or ((review.ab. and review.pt.) not trial.ti.) or ("we searched".ab. and (review.ti. or review.pt.)) or "update review".ab. or (databases adj4 searched).ab. or ((rat or rats or mouse or mice or swine or porcine or murine or sheep or lambs or pigs or piglets or rabbit or rabbits or cat or cats or dog or dogs or cattle or bovine or monkey or monkeys or trout or marmoset$1).ti. and animal experiment/) or (Animal experiment/ not (human experiment/ or human/)))

53 51 and 52

**Medline:**

1. exp Fibromyalgia/

2. fibromyalgia.mp.

3. fibromyalgia syndrome.mp.

4. fibrositis.mp.

5. fibromyositis.mp.

6. exp Exercise/

7. exercise.mp.

8. exp Exercise Therapy/

9. exercise therap*.mp.

10. Therapeutic exercise*.mp.

11. aerobic exercise*.mp.

12. aerobic training.mp.

13. exp Resistance Training/

14. resistance training.mp.

15. strength training.mp.

16. muscle training.mp.

17. muscle strengthening.mp.

18. strengthening exercise*.mp.

19. exp Weight Lifting/

20. weight lifting.mp.

21. resistance exercise*.mp.

22. exp Hydrotherapy/

23. hydrotherap*.mp.

24. aquatic exercise*.mp.

25. aquatic therap*.mp.

26. underwater exercise*.mp.

27. exp Muscle Stretching Exercises/

28. stretching exercise*.mp.

29. muscle stretching exercises.mp.

30. flexibility training.mp.

31. exp Yoga/

32. yoga.mp.

33. yoga therap*.mp.

34. therapeutic yoga.mp.

35. yoga pose*.mp.

36. yoga posture*.mp.

37. yoga asana*.mp.

38. exp Tai Ji/

39. Tai Ji.mp.

40. Tai chi.mp.

41. exp Qigong/

42. Qigong.mp.

43. physiotherap*.mp.

44. physical therap*.mp.

45. exp Rehabilitation/

46. rehabilitation.mp.

47. 1 or 2 or 3 or 4 or 5

48. 6 or 7 or 8 or 9 or 10 or 11 or 12 or 13 or 14 or 15 or 16 or 17 or 18 or 19 or 20 or 21 or 22 or 23 or 24 or 25 or 26 or 27 or 28 or 29 or 30 or 31 or 32 or 33 or 34 or 35 or 36 or 37 or 38 or 39 or 40 or 41 or 42 or 43 or 44 or 45 or 46

49. 47 and 48

50. ((randomized controlled trial or controlled clinical trial).pt. or randomized.ab. or randomised.ab. or placebo.ab. or drug therapy.fs. or randomly.ab. or trial.ab. or groups.ab.) not (exp animals/ not humans.sh.)

51. 49 and 50

52. limit 51 to (english language and humans)

**AMED**

1. exp Fibromyalgia/

2. fibromyalgia.mp.

3. fibromyalgia syndrome.mp.

4. fibrositis.mp.

5. fibromyositis.mp.

6. 1 or 2 or 3 or 4 or 5

7. exp Yoga/

8. yoga.mp.

9. yoga therap*.mp. [mp=abstract, heading words, title]

10. therapeutic yoga.mp. [mp=abstract, heading words, title]

11. yoga pose*.mp. [mp=abstract, heading words, title]

12. yoga posture*.mp. [mp=abstract, heading words, title]

13. yoga asana*.mp. [mp=abstract, heading words, title]

14. exp Tai chi/

15. Tai chi/ or Tai chi.mp.

16. exp Qigong/

17. Qigong.mp.

18. 7 or 8 or 9 or 10 or 11 or 12 or 13 or 14 or 15 or 16 or 17

19. 6 and 18

**CINAHL**

1. Fibromyalgia (MH^+^)
2. Fibromyalgia (TI OR AB)
3. Fibromyalgia syndrome (TI OR AB)
4. Fibromyositis (TI OR AB)
5. Exercise (MH^+^)
6. Aerobic exercise
7. Aerobic training
8. Resistance training (MH^+^)
9. Resistance exercise
10. Weight lifting (MH^+^)
11. Muscle strengthening (MH^+^)
12. Exercise therap*
13. Therapeutic exercise
14. Hydrotherapy (MH^+^)
15. pool based exercise*
16. Aquatic therapy (MH^+^)
17. Aquatic exercise
18. Stretching (MH^+^)
19. muscle stretching exercise*
20. flexibility exercise*
21. flexibility training
22. Yoga (MH^+^)
23. Yoga Asana*
24. Yoga Posture*
25. Tai Chi (MH^+^)
26. Qigong (MH^+^)
27. Physical Therapy (MH^+^)
28. Rehabilitation (MH^+^)
29. 1 OR 2 OR 3 OR 4
30. 5 OR 6 OR 7 OR 8 OR 9 OR 10 OR 11 OR 12 OR 13 OR 14 OR 15 OR 16 OR 17 OR 18 OR 19 OR 20 OR 21 OR 22 OR 23 OR 25 OR 25 OR 26 OR 27 OR 28
31. 29 AND 30

**SPORTS DISCUSS**

1. Fibromyalgia
2. Fibromyalgia syndrome
3. Fibromyositis
4. Exercise
5. Aerobic exercise
6. Aerobic training
7. Resistance training
8. Resistance exercise
9. Weight lifting
10. Muscle strengthening
11. Exercise therap*
12. Therapeutic exercise
13. Hydrotherapy
14. pool based exercise*
15. Aquatic therapy
16. Aquatic exercise
17. Stretching
18. muscle stretching exercise*
19. flexibility exercise*
20. flexibility training
21. Yoga
22. Yoga Asana*
23. Yoga Posture*
24. Tai Chi
25. Qigong
26. Physical Therapy
27. Rehabilitation (MH^+^)
28. 1 OR 2 OR 3 OR
29. 4 OR 5 OR 6 OR 7 OR 8 OR 9 OR 10 OR 11 OR 12 OR 13 OR 14 OR 15 OR 16 OR 17 OR 18 OR 19 OR 20 OR 21 OR 22 OR 23 OR 25 OR 25 OR 26 OR 27 OR 28
30. 29 AND 30

**COCHRANE:**

1. Exercise (MESH EXP)
2. Rehabilitation (MESH EXP)
3. Physical Therapy Modalities (MESH EXP)
4. Exercise therapy (MESH EXP)
5. Exercise (TI, AB, KEY)
6. Rehabilitation (TI, AB, KEY)
7. Physical therap* (TI, AB, KEY)
8. Physiotherap* (TI, AB, KEY)
9. exercise therap* (TI, AB, KEY)
10. #1 OR #2 OR #3 OR #4 OR #5 OR #6 OR #7 OR #8 OR #9
11. Fibromyalgia (MESH EXP)
12. Fibromyalg* (TI, AB, KEY)
13. Fibromyositis (TI, AB, KEY)
14. Muscular rheumatism (TI, AB, KEY)
15. #11 OR # 12 # OR 13 OR #14
16. #10 AND #15
17. Clinical trial (MESH EXP)
18. Randomized controlled trial* (MESH EXP)
19. Clinical trial* (TI, AB, KEY)
20. Randomi?ed controlled trial* (TI, AB, KEY)
21. Randomi?ed control trial* (TI, AB, KEY)
22. Random (TI, AB, KEY)
23. RCT (TI, AB, KEY)
24. #17 OR #18 19 #20 OR #21 OR 22 OR 23 OR 14
25. #16 AND #24

**Appendix2: Table: Summary of Data Extraction items**

| Author, Year | Baseline Characteristics | Details of Intervention | Details of Comparator | Pain Outcome & Mean Difference 95%(CI) | Mechanism proposed | Exclusion Criteria that may impact pain outcomes |
| --- | --- | --- | --- | --- | --- | --- |
| Acosta-Gallego et al. 2018 | Aquatic exercise:  Age: 58.05 (6.58)  Sex: 37 women  BMI: 28.28 (5.7)  Race: NR  Duration of symptoms: NR  Baseline Pain: 6.7(1.8)  Land based exercise:  Age: 58.25 (7.12)  Sex: 36 women  BMI: 27.03 (4.79)  Race: NR  Duration of symptoms: NR  Baseline Pain: 6.0 (1.8) | Type: Land-Based: 10 min. warm up (primarily based on walking), 25 min. exercise based on aerobic exercise, proprioception, balance and breathing. 10 min. stretching and relaxation.  Frequency: 2/week  Intensity: ASCM recommendations Principal of progressive load increase. Patients never attained high intensity workloads.  Duration: 50 min. sessions, 20 weeks | Type: Aquatic:  Frequency, intensity, duration and the components of the program are same as land based | VAS  Within Group (AG): -1 (-1.9 to -0.09)  Within Group (LG): 0.2 (-0.6 to 1.04)  Between Group:  0.5 (-0.41 to 1.41) | Buoyancy  Relaxation | History of fracture; Ingestion of drugs which can affect neuromuscular function |
| Swar, 2020 | Aquatic exercise:  Age: 52.57 (2.31)  Sex: 30 women  BMI: 28.37(1.06)  Race: NR  Duration of symptoms: NR  Baseline Pain: 8(0.98)  Land based exercise:  Age: 53.43 (2.70)  Sex: 30 women  BMI: 27.89 (1.30)  Race: NR  Duration of symptoms: NR  Baseline Pain: 8.07(0.91) | Type: Aquatic exercise: 5 min. warming up, 20 min. aerobic, strengthening and flexibility for the upper and lower limbs, and trunk, and 5 min. cool down. Cardiovascular exercises were bicycling simulation, walk around pool, continuous rhythmic activity and aerobics dance. Strength exercise was performed at slow pace by water and aquatic substances.  Frequency:2x/week  Intensity: Bodyweight and resistance bands at different degrees of resistance as the patient's ability permitted  Duration: 8 weeks | Type: Land based Combined Exercises: 5 min. warming up, 20 min. aerobic, strengthening and flexibility for the upper and lower limbs, and trunk, and 5 min. cool down. Cardiovascular exercises incorporated walk with many speeds and continuously and rhythmical activity which had affected largely on muscles and aerobics dance. Resistance strength training consisted of 1-3 sets of 8-12 reps. Flexibilities were developed by static stretching.  Frequency: 2/week  Intensity: Bodyweight and resistance bands at different degrees of resistance as the patient's ability permitted  Duration: 8 weeks | VAS  Within Group (AG): -2.07(-2.68 to -1.45)  Within Group (LG): -0.54(-1.02 to -0.05)  Between Group:  1.6 (0.98 to 2.21) | Exercise induced hypoalgesia | Use of analgesic or corticosteroid medications; BMI >30kg/m^2^; history of bone disease |
| Altan et al. 2004 | Aquatic exercise:  Age: 43.14 (6.39)  Sex: 24 women  BMI: NR  Race: NR  Duration of symptoms: NR  Baseline Pain (VAS): 7.91(1.81)    Balneotherapy:  Age: 43.91 (6.26)  Sex: 22 women  BMI: NR  Race: NR  Duration of symptoms: NR  Baseline Pain: 7.5(1.82) | Type: Aquatic Exercise (35degrees Celsius, Group): warm up (walking back and forth in the pool), activity (jumping in the pool and AROM and stretching of the neck and extremities), relaxation (lying supine on the water and slow swimming), and out-of-pool exercises (bending back and forth, squatting, and relaxing with deep breaths)  Frequency: 3x/week  Intensity: NA  Duration: 35 min; 12weeks | Type: Balneotherapy: Therapeutic minerals in the pool.  Frequency: 3/week  Intensity: NA  Duration: 12 weeks | VAS Pain, 5-point categorical pain Scale, Algometer score, Myalgic score  VAS:  Within Group (AG): -2.52 (-3.9 to -1.13)  Within Group (BG): -1.14 (-2.41 to 0.13)  Between Group:  0.97 (-0.58 to 2.52)  (Week 24 – Week 0) | Promotes Exercise induced hypoalgesia  Breaks Pain-immobility-Pain cycle  Prevents Muscular hypoxia | Unclear exclusion criteria |
| Altan et al. 2009 | Pilates group:  Age: 48.20 (6.5)  Sex: 25 women  BMI: NR  Race: NR  Duration of symptoms: NR  Baseline Pain: 6.1(1.7)  Home exercise program:  Age: 50.0 (8.4)  Sex: 24 women  BMI: NR  Race: NR  Duration of symptoms: NR  Baseline Pain: 6.3 (1.8) | Type: Pilates: 9 modules: postural education, search for neutral position, sitting exercise, antalgic exercises, stretching exercises, proprioception improvement exercises, and breathing education  Frequency: 3/week  Intensity: NA  Duration: one hour; 12 weeks | Type: Home exercise relaxation/stretching program: Relaxation techniques, dynamic (slow, controlled leg and arm swings), active stretching (i.e., bringing the leg up high and holding it there without anything to keep it in that extended position), and passive stretching (i.e., reaching to the feet when sitting up)  Frequency: 3/week  Intensity: Bodyweight  Duration: 1 hour; 12 weeks | VAS Pain, Algometric score (kg/cm^2^)  Within Group (PG): -2 (-2.96 to -1.03)  Within Group (HG): -0.3 (-1.43 to 0.83)  Between Group:  1.9 (0.8 to 2.9) | Improves Sleep  Promotes Exercise induced hypoalgesia  Breaks Pain-immobility-Pain cycle  Prevents Muscular hypoxia  Placebo effects  (supervision)  Mental conditioning | Use of Non-steroidal anti-inflammatory drugs  . |
| Andrade et al. 2019 | Aquatic exercise:  Age: 48 (8)  Sex: 27 women  BMI: NR  Race: NR  Duration of symptoms: NR  Baseline Pain: 5.8(2.7)  Control group:  Age: 47 (8)  Sex: 27 women  BMI: NR  Race: NR  Duration of symptoms: NR  Baseline Pain: 5.5(2.1) | Type: Aquatic exercise (heated pool at 30^°^ Celsius):  Frequency: 2/week  Intensity: NR  Duration: 32 sessions (16 weeks) | Control group:  Type: Maintenance of daily life activities identified at baseline | VAS; PPT; SF-36 bodily pain  SF 36:  Within Group (AG): -5.9(-14.5 to 2.7)  Within Group (CG): 3.7 (-2.61 to 10.01)  Between Group:  : -8.5 (-16 to -0.99) | Muscle oxygenation  Aquatic environment | Any other musculoskeletal, neurological, and rheumatic diseases |
| Assis et al. 2006 | Land based exercise:  Age: 42.17 (10.05)  Sex: 30 women  BMI: 26.89 (4.62)  Race: NR  Duration of symptoms (mo.): 83.12(64.84)  Baseline Pain:  Median (IQR)  7.5(6.75 -10).  Aquatic exercise:  Age: 43.43 (10.76)  Sex: 30 women  BMI: 27.31 (5.43)  Race: NR  Duration of symptoms (mo.): 61.93(47.17)  Baseline Pain:  Median (IQR)  8.0(8.0 -10). | Type: Deep Water Running: 10 min. stretching warm-up, 10 min. aerobic training, 10 min. relaxation  Frequency: 3/week  Intensity: heart rate at anaerobic threshold  Duration: 60 min; 15 weeks | Type: Land based exercise: 10 min stretching warm-up; aerobic training 40 min; 10 min relaxation  Frequency: 3/week  Intensity: heart rate at anaerobic threshold  Duration: 60 min; 15 weeks | VAS Pain  #Mean scores not reported  Statistical significance was ascertained based on p values; | Tissue oxygenation  Aquatic environment  Relaxation | Any other rheumatic diseases |
| Assumpcao et a. 2017 | Stretching group:  Age: 47.9 (5.3)  Sex: 14 women  BMI: 28.9 (4.2)  Race: NR  Duration of symptoms: NR  Baseline Pain: 5.6 (1.8)  Resistance group:  Age: 45.7 (7.7)  Sex: 16 women  BMI: 28.1 (4.7)  Race: NR  Duration of symptoms: NR  Baseline Pain:  5.3 (2.5)  Control group:  Age: 46.9 (6.5)  Sex: 14 women  BMI: 29.4 (4.8)  Race: NR  Duration of symptoms: NR  Baseline Pain: 6.0 (2.6) | Type: Stretching: Triceps sura, gluteus, ischio-tibial, paravertebral, latissimus dorsi, hip adductor, and pectoralis muscles  Frequency: 2x/week  Intensity: 30s stretch at moderate discomfort  Duration: 40 min; 12 weeks | Type: Resistance exercise: triceps surae, quadriceps, hip adductors and abductors, hip flexors, elbow flexors and extensors, pectoralis major, and rhomboids  Frequency: 2x/week  Intensity: In the first two sessions, no load was used.0.5 kg was added each week if the patient identified the effort as slightly intense on the Borg scale (score = 13)  Duration: 40 min; 12 weeks  Type: No Intervention Control group  Frequency: NA  Intensity: NA  Duration: NA | VAS Pain,  Pain threshold (PPT),  FIQ Pain,  SF-36 Bodily Pain  Only, SF-36 bodily pain is significantly different between the groups.  Mean values are not reported; statistical significance determined based on p values | Improves freedom of movement | Noncontrolled systemic disorders, neurological and musculoskeletal conditions, relevant joint disorders. |
| Atan et al.,  2020 | HIIT:  Age: 46.57 (9.41)  Sex: 19 females  BMI: 31.1  Race: NR  Duration of symptoms (mo): 37.57 (21.52)  Baseline Pain:  7.78(1.81)  MICT:  Age: 47.36(8.01)  Sex: 19 females  BMI: 31.0  Race: NR  Duration of symptoms: 24 (14.96)  Baseline Pain:  7.89(1.76)  Control:  Age: 52.70 (8.96)  Sex: 17 females  BMI: 32.1  Race: NR  Duration of symptoms: 27.82(1.66)  Baseline Pain:  8.29(1.61) | Type: Combined exercise  HIIT plus stretching and strengthening:  5 min warm up cycling 4 sets; 4 min HIIT 3 sets; 3 min active recovery; 5 min cool down.  15 min of stretching and strengthening  shoulder press, dumbbell press, shoulder elevation with resistance, biceps curl, squats, hip flexion, and extension, and standing hip exercises using 1-3 kg of weight loads and 1 set of 8-10 repetitions stretching exercises were performed for 5 minutes by holding the main muscle-tendon groups for 20-30 seconds, with 4-5 repetitions for each muscle group.  Frequency: 5/week  Intensity: 5 min. warm up cycling (50% peak HR), followed by 4 sets of 4 min (80-95% pear HR), interspersed with 3 sets of 3 min active recovery (70% peak HR), 5 min. cool down (50% peak HR) Strengthening: 1-3kg, and 1 set of 8-10 reps Stretching: 20-30 seconds with 4-5 reps for each muscle group  Duration: 35 min; 6 weeks (30 sessions) | Type: Combined exercise  MICT plus stretching and strengthening same as HIIT:  5 min warm up cycling, continuous activity, and cool down period.  Frequency: 5/week  Intensity: Aerobic: 5 min. warm up (50% peak HR), continuous activity (65-70% peak HR) and 5 min. cool down (50% peak HR). Strengthening: Stretching: same as HIIT group  Duration: 55 min; 6 weeks (30 sessions)  Control group:  Type: Did not participate in any supervised exercise sessions, but they had specific recommendations regarding exercise for FM.  Frequency: NA  Intensity: NA  Duration: NA | VAS Pain, SF-36 Pain  VAS:  Within Group (HIIT group):  -3.57 (-4.8 to -2.33)  Within Group (MICT group):  -3.53(-4.92 to -2.13)  Within Group (CG):  -0.41 (-1.45 to 0.63)  Between Group:  (HIIT vs MICT):  -0.13 (-1.40 to 1.14)  Between Group:  (HIIT vs C):  -2.08 (-3.39 to -0.77)  Between Group:  (MICT vs C):  -1.95 (-3.26 to – 0.64) | Maintain healthy body composition values | Analgesic drugs, and/or connective tissue diseases and/or musculoskeletal problems |
| Britto et al. 2020 | Aquatic exercise group:  Age: 50.25 (6.09)  Sex: 16 females  BMI: NR  Race: NR  Duration of symptoms: NR  Baseline pain:  7.82(2.05)  Land based exercise group:  Age: 46.18 (10.84)  Sex: 17 females  BMI: NR  Race: NR  Duration of symptoms: NR  Baseline pain: 8.76 (1.41) | Type: Aquatic exercise (33^°^ Celsius): 10 min warm-up, 10 min. active stretching, 30 min. strengthening, 10 min. relaxation  Frequency: 3/week  Intensity: "training intensities were similar in the two groups"  Duration: 60 min; 8 weeks | Type: Land based exercise:  Same as aquatic exercise  Frequency: 3/week  Intensity: "training intensities were similar in the two groups"  Duration: 60 min; 8 weeks | VAS Pain, FIQ Pain  VAS:  Within Group (AG):  -1.32 (-3.13 to 0.49)  Within Group (LG):  -1.08 (-2.54 to 0.38)  Between Group:  -0.1(-1.77 to 1.57)  P-value significant^*^. | Aquatic environment  Muscle relaxation  Self esteem | Structural musculoskeletal deformities, herniated discs, and rheumatoid arthritis |
| Da Silva et al. 2007 | Yoga group:  Age: 46.3 (8.9)  Sex: 17 females  BMI: NR  Race: Caucasian: 12 Mixed: 3 Black: 1 Asian: 1  Duration of symptoms: NR  Baseline Pain: NR  Yoga and Touch group:  Age: 44.4 (11.0)  Sex: 16 females  BMI: NR  Race: Caucasian: 11 Mixed: 3 Black: 1 Asian: 1  Duration of symptoms: NR  Baseline Pain: NR | Type: Relaxing Yoga: started with simple postures of stretching involving all movements of the vertebral column. Next, 7 minutes of diaphragmatic yogic breathing when laying in a cot. Next, 15 min. relaxation technique focusing the subject's attention sequentially on each major body segment.  Frequency: 1/week  Intensity: NA  Duration: 50 min; 8 weeks | Type: Relaxing Yoga plus Touch: Same as Group 1, but with external touch during the relaxation phase  Frequency: 1/week  Intensity: NA  Duration: 50 min; 8 weeks  . | VAS Pain  Mean values are not reported; statistical significance determined based on reported p values | Reduces muscle tension  Promotes Relaxation  Reduces Sympathetic hyperactivity | Unclear exclusion criteria  . |
| de Andrade et al. 2008 | Pool based Aquatic exercise group:  Age: 48.8 (9.9)  Sex: 23 females  BMI: NR  Race: NR  Duration of symptoms: NR  Baseline Pain: 9.4(1.1)  Sea based exercise group:  Age: 48.3 (8.9)  Sex: 23 females  BMI: NR  Race: NR  Duration of symptoms: NR  Baseline Pain: 9.1(1.1) | Type: Pool based (28-33^°^ Celsius): 10 min. stretching, 40 min. low-impact aerobic exercise, 10 min. relaxation period  Frequency: 3/week  Intensity: First 2 weeks: 10-11 on BORG 3rd to 12th weeks: 12-13 on BORG  Duration: 60 min; 12 weeks | Type: Sea based  Same as pool based  Frequency: 3/week  Intensity: First 2 weeks: 10-11 on BORG 3rd to 12th weeks: 12-13 on BORG  Duration: 60 min; 12 weeks | VAS Pain  Within Group (AG):  -3.6 (-4.59 to -2.60)  Within Group (SG):  -3.7 (-4.58 to -2.81)  Between Group:  0.4 (-0.76 to 1.56) | Effects of Aquatic environment | Any other rheumatic diseases |
| de Medeiros et al. 2020 | Aquatic aerobic exercise group:  Age: 50.7 (9.7)  Sex: 20 females  BMI: 30.4 (5.2)  Race: NR  Duration of symptoms: NR  Baseline Pain: 7.5(1.8)  Mat Pilates group:  Age: 45.5 (10.6)  Sex: 21 females  BMI: 27.8 (4.7)  Race: NR  Duration of symptoms: NR  Baseline Pain: 7.5(1.6) | Type: Mat Pilates:  Nine exercises were performing for the main muscle groups. Exercises were initially performed in 1 series of 8 repetitions in the first month, 2 sets of 10 repetitions in the second month, and 3 sets of 8 repetitions in the last month. Three Swiss ball relaxation exercises performed in 1 set of 30 seconds at the end of each session.  Frequency: 2x/week  Intensity: variable intensities (Borg scale)  Duration: 40 min; 12 weeks | Type: Aquatic Aerobic Exercise (31^°^ Celsius): Six main exercises, 2 warm-up exercises, and 2 cool-down exercises  Frequency: 2x/week  Intensity: variable intensities (Borg scale)  Duration: 50 min; 12 weeks | VAS Pain, SF-36 Pain  VAS  Within Group (AAEG):  -1.9 (-3.22 to -0.57)  Within Group (MPG):  -1.3 (-2.23 to -0.36)  Between Group:  -0.61 (-1.8 to 0.6) | Inhibition of pain in CNS  Aquatic environment | Decompensated diabetes, severe psychiatric illness, history of regular exercise. |
| Evcik et al. 2008 | Aquatic exercise group:  Age: 43.8 (7.7)  Sex: 31 females  BMI: 25.6 (3.8)  Race: NR  Duration of symptoms (yrs.):  3(2.3)  Baseline Pain: 6.2(1.7)  Home based exercise group:  Age: 42.8 (7.6)  Sex: 30 females; 1 male  BMI: 27.9 (5.1)  Race: NA  Duration of symptoms: 3(1.9)  Baseline Pain: 6.1(1.9) | Type: Aquatic Exercise (33^°^ Celsius): 20 min. poolside warmup, AROM and relaxation, followed by 35 min. of aquatic exercises including walking backwards and forwards in the pool, aerobic exercises such as jumping and jogging, active ROM, stretching of neck and the extremities, and relaxation such as lying supine and low impact swimming. Lastly, a 5 min. cooldown.  Frequency: 3/week  Intensity: NA  Duration: 60 min; 5 weeks | Type: Home-Based Exercise:  warmup, ROM, relaxation, aerobic, stretching, and cool down  Frequency: 3x/week  Intensity: NA  Duration: 60 min; 5 weeks | VAS Pain  Within Group (AAE):  -2.3(-3.21 to -1.38)  Within Group (HBE):  -1 (-2.01 to 0.01)  Between Group:  1.2 (0.18 to 2.2); AAE is superior | Promotes Exercise induced hypoalgesia | Nonsteroidal anti-inflammatory drugs, regular exercise habitation, inflammatory joint diseases. |
| Garcia-Martinez et al. 2012 | Mixed exercise group:  Age: 59.3 (4.8)  Sex: 14 females  BMI: 27.6 (4.3)  Race: NR  Duration of symptoms: 9.9(3.8)  Baseline Pain: 26.7(21.4)  Control group:  Age: 58.6 (7.8)  Sex: 14 females  BMI: 29.0 (4.9)  Race: NR  Duration of symptoms: 10.6(4.1)  Baseline Pain: 34.6(15.0) | Type: Combined Exercise:  10 min. warm up with slow walks and easy movement, 20 min. aerobic exercise (starting at 60-70% HRmax and increased to 75-85% HRmax), 20 min. stretching and strength exercise, and 10 min. cooldown  Frequency: 3/week  Intensity: Aerobic exercise starting at 60-70% HRmax and increased to 75-85% HRmax  Duration: 60 min; 12 weeks | No treatment control group:  Type: subjects continued their daily activities, which did not include any physical exercise similar to those in the program  Frequency: NA  Intensity: NA  Duration: NA | SF-36 Bodily Pain  Within Group (CE):  20.8(4.01 to 37.58)  Within Group (CG):  -9.4(-20.26 to 1.46)  Between Group:  -22.3(-36.21 to -8.38) | Belonging to Group  Self-efficacy  Psychological distress and Mental health | Any other inflammatory rheumatic disease, participation in a physical therapy or exercise program in the last 6 months. |
| Gavi et al. 2014 | Group1:  Age: 44.34 (7.94)  Sex: 35 females  BMI: 26.0  Race: NR  Duration of symptoms: NR  Baseline Pain:  7.81(1.59)  Group2:  Age: 48.65 (7.60)  Sex: 31 females  BMI: 27.8  Race: NR  Duration of symptoms: NR  Baseline Pain:  8.38(1.46) | Strengthening exercise group:  Type: Progressive training in standing and sitting using weight machines. Muscle groups trained: quadriceps femoris, hamstrings, biceps brachii, triceps brachii, pectoral, calf, deltoid, and latissimus dorsi  Frequency: 2/week  Intensity: 45% of 1RM; 3 sets of 12 reps  Duration: 45 min; 16 weeks | Flexibility group  Type: Stretching of quadriceps femoris, hamstrings, biceps brachii, triceps brachii, pectoral, calf, deltoid, and latissimus dorsi  Frequency: 2/week  Intensity: NA  Duration: 45 min; 16 weeks | VAS Pain; SF-36 Bodily pain  For VAS, mean values are not reported; statistical significance is determined based on p values  For SF-36  Within Group (SEG):  15 (8.24 to 21.75)  Within Group (STG):  12.9(5.2 to 20.69)  Between Group:  -0.19(-7.96 to 7.58) | Promotes autonomic modulation | Any other rheumatic diseases, autonomic dysfunction, opioid analgesics, exercise within the last 3 months. |
| Genc et al. 2015 | Home exercise group:  Age: 36.9  Sex: 25 females  BMI: NR  Race: NR  Duration of symptoms (mo.): 62.2  Baseline Pain:  73.96(23.47)  Aerobic exercise group:  Age: 35.1  Sex: 25 females  BMI: NR  Race: NR  Duration of symptoms (mo.): 67.4  Baseline Pain:  76.04(20.07) | Type: Aerobic Exercise: Home exercise program + treadmill walking  Frequency: 3x/week  Intensity: 60-75% of HRmax for aerobic  Duration: 6 weeks | Frequency: 2x/day  Intensity: NA  Duration: 6 weeks  Type: Home Exercises: Flexibility and stretching (trunk, hips, ankles, shoulders, wrist, neck, lateral side, chest, shoulder, arms, triceps, quadriceps, calves, and hamstrings) | VAS Pain, SF-36 Bodily Pain  VAS (0-100):  Within Group (HE):  -10(-22.14 to 2.14)  Within Group (AE):  -20.48(-31.89 to -9.06)  Between Group:  -8.4(-19.52 to 2.72) | Positive emotional effects  Endorphins  HPA axis (hormones)  Improving serum cortisol, ACTH, GH, IGF-1 | Any other connective disorders, orthopedic disorder, psychological or physical therapy in the last 3 months, in need of medication for anxiety and depression |
| Gusi et al. 2006 | Aquatic exercise group:  Age: 51 (10)  Sex: 17 females  BMI: 27 (5)  Race: NR  Duration of symptoms (yr.):  24(9)  Baseline Pain:  63.1(26.0)  Control group:  Age: 51 (9)  Sex: 17 females  BMI: 27 (4)  Race: NR  Duration of symptoms:  19(8)  Baseline Pain:  63.9(25.0)  : | Type: Aquatic Exercise (33^°^ Celsius): 10 min. warmup (slow walks and mobility exercises), 10 min aerobic exercise (65-75% HRmax), 20 min. overall mobility and lower limb strength (4x10 reps of knee flexion and extension with body vertical using water as resistance), another 10 min. of aerobics (65-75% HR max), and 10 min. cool down with low-intensity exercises.  Frequency: 3/week  Intensity: aerobic: 65-75% HR max.  Duration: one hour; 12 weeks | No treatment Control:  Type: continued to follow normal daily activities, which did not include any form of exercise related to those in therapy.  Frequency: NA  Intensity: NA  Duration: NA | VAS Pain (0-100)  Within Group (AG):  -18.4 (-31.5 to -5.3)  Within Group (CG):  1.0(-7.2 to 9.3)  # Could not calculate Between Group  scores are post mean values are not reported; and only the post-pre changes scores are reported as above | Effects of aquatic environment | Severe disorders of spine, history of trauma, frequent migraines, history of more than one 30-minutes exercise session per week during 2 weeks in the last 5 years |
| Hernando-Garijo et al. 2021 | Aerobic exercise group:  Age: 51.81 (9.05)  Sex: 17 women  BMI: 27.25 (7.30)  Race: NR  Duration of symptoms: NR  Baseline pain: 7.08(1.45)  Control group:  Age: 55.06 (8.51)  Sex: 17 women  BMI: 25.93 (5.27)  Race: NR  Duration of symptoms: NR  Baseline pain:  7.29(1.05) | Type: Telerehabilitation Aerobic Program:  Low impact rhythmic movements with warm-up (joint mobility exercises and active stretching, central part (aerobic exercises based on low-impact rhythmic movements), and cool-down (static stretching and breathing techniques).  Frequency: 2x/week  Intensity: Moderately intense aerobic exercise  Duration: 15 weeks | No treatment control group  Type: No additional interventions  Frequency: NA  Intensity: NA  Duration: NA | VAS; Algometer score  Within Group (AG):  2.15(1.37 to 2.94)  Within Group (CG):  0.82(-0.3 to 1.68)  Between Group:  1.54(0.17 to 2.9) | HPA function  Neurotransmitters  Endorphins | Psychiatric disorders |
| Hooten et al. 2012 | Strength group:  Age: 47.3 (10.1)  Sex: 3 men; 33 women  BMI: NR  Race: 100% White  Duration of symptoms: 13.4(14.9)  Baseline pain: 46.4(9.8)  Aerobic group:  Age: 45.8 (11.5)  Sex: 4 men; 32 women  BMI: NR  Race: 92% White 2% Hispanic 2% Arabic 2% African American  Duration of symptoms: 11.5(10.9)  Baseline pain: 48.6(6.7) | Type: Strengthening: Warm-up, strengthening exercises (biceps femoris, quadriceps, and biceps brachii) and cooldown  Frequency: daily  Intensity: 1 set of 10 reps at individually specified weight loads 1-3 kg for UE; 3-5 kg for LE  Duration: 25 to 30 min; 3weeks | Type: Aerobic:  Warm-up, Stationary bicycle, cooldown  Frequency: Up to 10 min daily (50 min total) during the first week, up to 15 min daily (1.25-hour total) during the second week, and up to 30 min. daily (2.5-hour total) during the third week.  Intensity: Participants were encouraged to gradually increase the intensity and duration of aerobic exercise to achieve 70%-75% of HRmax based on age  Duration: 20-30 min; 3 weeks | Pressure pain threshold, Pain Severity using Multidimensional pain inventory  MDPI:  Within Group (SG):  -12 (-17.02 to -6.99)  Within Group (AG):  11 (-15.53 to -6.46)  Between Group:  -2.2 (-6.1 to 1.7) | Reduction in peripheral and central sensitization  Muscle oxygenation | Any other orthopedic or systemic diseases |
| Izquierdo-Alventosa et al. 2021 | Mixed exercise group:  Age: 53.06 (8.40)  Sex: 16 women  BMI: NR  Race: NR  Duration of symptoms: 8.47 (6.32)  # Mean of all there groups  Baseline Pain: 6.13 (2.22)  TMS group:  Age: 50.47 (8.90)  Sex: 17 females  BMI: NR  Race: NR  Duration of symptoms: see above  Baseline pain: 6.18(2.01)  Control group:  Age: 55.13 (7.35)  Sex: 16 females  BMI: NR  Race: NR  Duration of symptoms: see above  Baseline pain: 5.63(1.75) | Type: Combined exercise group:  Low-intensity physical exercise. 10 min. warm-up, 40 min. training (endurance and coordination), and 10 min. cooldown.  Frequency: 2x/week  Intensity: weak to moderate effort on Borg scale  Duration: 60-min; 2weeks | TMS group:  Type: TMS targeting M1 location  Frequency: 5/week  Intensity: NA  Duration: 20 min; 2 weeks  Control group:  Type: No therapy assigned; participants were asked to perform their usual routines.  Frequency: NA  Intensity: NA  Duration: NA | VAS Pain, Average PPT  PPT:  Within Group (CE):  0.32(-0.4 to 1.04)  Within Group (TMS):  0.72(-0.46 to 1.9)  Within Group (CG):  -0.26(-0.62 to 0.1)  Between Group:  (CE vs TMS):  0.6(-0.53 to 1.73)  Between Group:  (CE vs CG):  -0.57(-1.17 to 0.03)  Between Group:  (TMS vs CG):  -0.57(-1.17 to 0.03)  # Reported p values as significant | Exercise induced hypoalgesia  Physical conditioning  Changes in Oxygen kinetics  HPA axis  Biomarkers (serotonin and dopamine) | Advanced MSK pathologies, history of intense headaches, previous exercise program in the last two months, psychiatric pathologies |
| Izquierdo-Alventosa et al. 2020 | Combined exercise group:  Age: 53.06 (8.4)  Sex: 16 women  BMI: NR  Race: NR  Duration of symptoms: NR  Baseline pain: NR  Control group:  Age: 55.13 (7.35)  Sex: 16 women  BMI: NR  Race: NR  Duration of symptoms: NR  Baseline pain: NR | Type: Combined Low-Intensity Physical Exercise Group: Stage 1(sessions 1-4) was devoted to participants' adjustment and familiarization with exercise. Stage 2 (sessions 5-16) were aimed at personalized strength and coordination training. Three parts of each session: warm-up (walking at slow pace and moving main joints), training (10 exercises with weights and elastic bands), and cooldown (walking at slow pace, overall trunk stretching, and breathing deeply while lying on the floor).  Frequency: 2/week  Intensity: Upper limbs: 0.5-2kg Lower limbs: 1-3kg 3-4 on the Borg scale (moderate effort)  Duration: 60 min; 16 sessions: 8 weeks | Control group:  Type: No intervention and patients were asked to perform their daily routines. Logbooks were used to record the type of physical activity undertaken and the approximated number of hours per week to ensure that no participant undertook intense physical activity.  Frequency: NA  Intensity: NA  Duration: NA | Pressure Pain Threshold  Within Group (CE):  0.32 (-0.40 to 1.04)  Within Group (CG):  -0.26(-0.62 to 0.10)  Between Group:  -0.57(-1.17 to 0.03)  # Reported p values as significant | Reduces Catastrophism  Distraction | Advances stage pathology (e.g., knee osteoarthritis), h/o intense headaches, exercise program in the last two months, h/o surgery in the last four months |
| Jablochkova et al. 2019 | Resistance exercise group:  Age: 50.8 (9.6)  Sex: 38 females  BMI: 27.4 (4.7)  Race: NR  Duration of symptoms: 9.6(6.5)  Baseline pain: 53.1(20.6)  Relaxation group:  Only reported mean values for both groups as a whole; see above  Sex: 34 females | Type: Resistance Exercise: 10 min. warm-up, 50 min. strength training focused on the lower body.  Frequency: 2x/week  Intensity: Program started at 40% maximum voluntary capacity (MVC) and increased to 70-80% MVC  Duration: 50 min; 15 weeks | Type: Relaxation Therapy: relaxation and autosuggestion  Frequency: 2x/week,  Intensity: NA  Duration: 25 min,  15 weeks | VAS Pain (0-100), PPT  VAS:  Within Group (RSG):  -12.3 (-22.97 to -1.62)  Within Group (RXG):  -3.20(-12.13 to 5.73)  Between Group:  16.8(6.44 to 27.15) | Improves Neurotransmitters  & Metabolic profile in muscle | Osteoarthritis in knee or hip, severe somatic or psychiatric disorders, causes of pain other than FM |
| Jentoft et al. 2001 | Aquatic exercise group:  Age: 42.9 (8.6)  Sex: 18 women  BMI: NR  Race: NR  Duration of symptoms (yr.): 11.1(5.7)  Baseline Pain: 6.9(1.7)  Land based exercise:  Age: 39.4 (8.8)  Sex:16 women  BMI: NR  Race: NR  Duration of symptoms (yr.): 11.1(8.4)  Baseline Pain:  5.8(2.0) | Type: Pool-based exercise (34^°^ Celsius): Body awareness training, ergonomics, warm-up exercises, aerobic dance, cooling down exercises, muscle stretching exercises, and relaxation training  Frequency: 2x/week  Intensity: 60-80% HRmax in 40-50% of the sessions  Duration: 60 min; 20 weeks | Type: Land-based exercise:  Same as aquatic group  Frequency: 2x/week  Intensity:  60-80% HRmax in 40-50% of the sessions  Duration: 60 min; 20 weeks | FIQ Pain, Exercise-induced pain  Within Group (AG):  -1.3(-2.67 to 0.07)  Within Group (LG):  -0.6(-2.48 to 1.28)  Between Group:  -0.4(-2.29 to 1.49)  #Reported significant p values for within group differences | Effects of aquatic environment | Any other inflammatory rheumatic diseases |
| Jones et al. 2002 | Resistance exercise group:  Age: 49.2 (6.36)  Sex: 28 women  BMI: NA  Race: White - 25 Non-White – 3  Duration of symptoms (yr.):  6.9(6.6)  Baseline Pain: 6.5(0.39)  Flexibility exercise group:  Age: 46.4 (8.56)  Sex: 28 women  BMI: NR  Race:  White - 26 Non-White – 2  Duration of symptoms: 7.7(5.5)  Baseline Pain: 6.15(0.36) | Type: Resistance exercise:  5 min. warm-up (marching and rhythmic dancing and gentle stretching), 45 min. muscle strengthening (gastrocnemius, tibialis anterior, quadriceps, hamstrings, gluteus, abdominals, erector spinae, pectorals, latissimus dorsi and rhomboids, deltoids, biceps and triceps), and 10 min. cooldown and stretching  Frequency: 2x/week  Intensity: 4 to 5 reps; progressing to 12 reps; progressively increase the resistance  Duration: 60 min; 12 weeks | Type: Flexibility training: Stretches targeted the same 12 muscle groups. Low intensity warmup of marching in place or rhythmic dance for 10 min., gentile stretching for 40 min., and guided imagery and relaxation for 10 min.  Frequency: 2/week  Intensity: Low intensity and gentle stretching  Duration: 60 min; 12 weeks | Total myalgic score; VAS with in the FIQ  Within Group (RG):  -1.89(-2.09 to -1.68)  Within Group (FG):  -1.01(-1.21 to -0.80)  Between Group:  0.53(0.31 to 0.74) | Promotes self-efficacy | Current participation in a regular exercise program |
| Kayo et al. 2012 | Walking group:  Age: 47.7 (5.3)  Sex: 30 women  BMI: 26.3 (4.5)  Race: NR  Duration of symptoms: 4.0(3.1)  Baseline Pain: 32.13(16.34)  Resistance exercise group:  Age: 46.7 (6.3)  Sex: 30 women  BMI: 26.2 (4.8)  Race: NR  Duration of symptoms (yr.): 4.7(5.7)  Baseline Pain:  26.60(14.62)  Control group:  Age: 46.1 (6.4)  Sex: 30 women  BMI: 26.7 (4.9)  Race: NR  Duration of symptoms (yr.): 5.4(3.5)  Baseline Pain:  31.40(13.07) | Type: Walking program: 5 min. warm-up period, 5-10 min. stretching, conditioning stimulus, and a cool down period.  Frequency: 3x/week  Intensity: Every 4 weeks, walking duration was increased (25-30 min. to 50 min.), as well as the intensity of the conditioning stimulus (beg at 40-50% ad progressed to 60-70% Reserve)  Duration: 60 min; 16 weeks | Type: Muscle Strengthening: 11 free active exercises, using free weights and body weight performed in the sitting, standing, and lying positions to improve strength of the upper and lower limbs, and trunk muscles.  Frequency: 3x/week  Intensity: according to patients’ tolerance on Borg scale 3 sets of 10 reps increased to 15 reps after week 5  Duration: 60 min; 16 weeks  Control group:  Frequency: NA  Intensity: NA  Duration: NA  Type: Control | VAS Pain, SF-36 Bodily Pain  #Mean values not reported; statistical significance is determined based on p value. | Reduces Deconditioning | None |
| Kurt et al. 2016 | Balneotherapy group:  Age: 38.06 (10.90)  Sex: 37 women  BMI: NR  Race: NR  Duration of symptoms: 5.45(2.66)  Baseline pain: NR  Balneotherapy plus exercise:  Age: 35.13 (11.60)  Sex: 36 women  BMI: NR  Race: NR  Duration of symptoms: 5.2(2.30)  Baseline pain: NR  Combined Exercise group:  Age: 41.94 (12.78)  Sex: 36 women  BMI: NR  Race: NR  Duration of symptoms: 6.23(1.37)  Baseline pain: NR | Type: Balneotherapy + Exercise: 42^°^ Celsius with therapeutic minerals. Aerobic exercise and Muscle stretching, strengthening and relaxation exercises  Frequency: 5x/week  Intensity: (60 to 70% increase in HR)  Duration: 15 sessions; 3 weeks | Type: Balneotherapy:  42^°^ Celsius with therapeutic minerals  Frequency: 5x/week  Intensity: NA  Duration: 15 sessions; 3 weeks  Type: Combined Exercise: Aerobic exercise and Muscle stretching, strengthening and relaxation exercises  Frequency: 5x/week  Intensity: (60 to 70% increase in HR)  Duration: 25 to 35 minutes; 3 weeks | Total myalgic score  Within Group (BT plus E):  54.2(25.20 to 83.19)  Within Group (BT):  93.05(65.66 to 120.43)  Within Group(E):  36.1(20.13 to 52.06)  Between Group:  BT+E vs BT  -33.4(-58.30 to -8.49)  Between Group:  BT+E vs CE  40.8(20.5 to61.09)  Between Group:  BT vs CE  -74.2(-94.94 to -53.35) | Promotes EIH  Breaks pain-immobility pain  Muscle hypoxia  Sleep quality | Psychiatric disorders, cancer, advanced osteoarthritis, joint malformation, spinal disorders, or trauma within the last three months, inflammatory rheumatic disorders, participation in a physical therapy program in the last year |
| Kutlu et al. 2020 | Exercise group:  Age: 39.44 (8.28)  Sex: 27 women  BMI: 26.1  Race: NR  Duration of symptoms: NR  Baseline Pain: 5.67(2.10)  Exercise and Vagal stimulation group:  Age: 38.60 (9.34)  Sex: 25 women  BMI: 25.8  Race: NR  Duration of symptoms: NR  Baseline Pain: 6.17(2.58) | Frequency: 5x/week  Intensity: 10 reps; 2 sets  Duration: 4 weeks  Type: Mixed exercise  strengthening, stretching, isometric, and posture exercises | Frequency: 5x/week  Intensity: NA  Duration: 30 minutes; 4 weeks  Type: Exercise + Vagus Stimulation: Exercise same as other group  , | VAS; SF-36 bodily pain  VAS:  Within Group (EG):  -2.22(-3.27 to -1.16)  Within Group (E plus VG):  -3.61(-4.9 to -2.31)  Between Group:  -0.89(-1.89 to 0.11) | Autonomic changes  Promotes exercise induced hypoalgesia  Sleep | Chronic inflammation, using new drugs in the last month |
| Larsson et al. 2015 | Resistance exercise group:  Age: 50.81 (9.05)  Sex: 67 women  BMI: 27.39 (5.29)  Race: NR  Duration of symptoms (yr.): 11.06(8.53)  Baseline Pain:  49.3(23.9)  Relaxation group:  Age: 52.10 (9.78)  Sex: 63 women  BMI: 28.66 (5.32)  Race: NR  Duration of symptoms: 9.44(7.33)  Baseline Pain:  52.4(18.3) | Type: Strengthening exercise  10-minute warm up 50 min strengthening leg-press, knee-extension and knee-flexion using weight machine, biceps curl and hand grip strength using free weights, heel raise and core stability using body weight and 10 minutes of stretching exercises  Frequency: 2/week  Intensity: 40% of 1 RM; 15-20 reps; 1-2 sets progressed to 60% of 1RM; 10-12 reps; 1-2 sets 80% of 1RM; 5-8 reps; 1-2 sets  Duration: 15 weeks | Type: Relaxation Therapy: Series of exercises for relaxation and autosuggestion  Frequency: 2/week  Intensity: NA  Duration: 25 mins; 15 weeks | VAS Pain (0-100)  Within Group (SG):  -10.7(-19.48 to -1.91)  Within Group (RG):  1(-6.19 to 8.19)  Between Group:  14.8(5.90 to 23.69) | Improves Pain acceptance | Osteoarthritis (OA) in hip or knee, severe somatic or psychiatric disorders, other dominating causes of pain than FM, participation in a rehabilitation program within the past year, within the past year, regular resistance exercise or relaxation exercise twice a week or more, and not being able to refrain from analgesics, non-steroidal anti-inflammatory drugs (NSAID) or hypnotic drugs for 48 hours prior to examinations. |
| Letieri et al. 2013 | Aquatic exercise:  Age: 58.2 (10.6)  Sex: 33 women  BMI: 28.3 (4.6)  Race: NR  Duration of symptoms: NR  Baseline Pain: 60.1(5.7)  Control group:  Age: 59.4 (9.4)  Sex: 31 women  BMI: 29.9 (3.4)  Race: NR  Duration of symptoms: NR  Baseline Pain: 59.8(50.6)  : | Type: Aquatic exercise (33^°^ Celsius, 1.30m depth): 5 min. warm-up exercises, 35 min. of exercise aimed to develop strength, mobility, balance, coordination, and agility, and 5 min. stretching and relaxation.  Frequency: 2/week  Intensity: Moderate intensity (60-70% HRmax)  Duration: 45 min; 15 weeks | Type: Instructed to refrain from any type of intervention or physical therapy during study period  Frequency: NA  Intensity: NA  Duration: NA | VAS Pain  #Mean values not reported; statistical significance was ascertained based on p values | Effects of Aquatic environment  Relaxation | disabling chronic clinical picture or associated chronic pain |
| Maddali Bongi et al. 2016 | Tai Ji group:  Age: 50.36 (13.68)  Sex: 22 Total  Males: Females not reported  BMI: NR  Race: NA  Control group:  Age: 54.30 (10.65)  Sex: 22 total  Males: Females not reported  BMI: NR  Race: NR | Type: Tai Ji Quan: 15 min. stage 1 (breathing exercises, concentration and postural maintenance), 15 min. stage 2 (very low impact movements allowing physical, mental and emotional rebalancing, thus improving greater control of posture, breathing and concentration), and stage 3 (a form of TJQ allowing the patient to carry out a precise movement that becomes more harmonious and consonant with the environment)  Frequency: 2x/week  Intensity:  Duration: 60 min; 16 weeks | Type: educational session in order to give information about the disease, symptoms management and the ability to cope with them.  Frequency: NA  Intensity: NA  Duration: NA | SF-36; bodily pain  Within Group (TG):  14.82(6.61 to 23.02)  Within Group (CG):  -1.36(-11.15 to 8.43)  Between Group:  -5.45(-15.27 to 4.37) | Improves vital energy  Relaxation  Improved sleep | None |
| Matsutani et al. 2007 | Laser and stretching group:  Age: 44(28-60)  Sex: 10 women  BMI: 25.9(4.7)  Race: NR  Duration of symptoms: NR  Baseline Pain: 7.3(2.6)  Stretching group:  Age: 45(31-57)  Sex: 10 women  BMI: 26.5(5.4)  Race: NR  Duration of symptoms: NR  Baseline Pain: 7.5(2.1) | Type: Laser therapy and stretching: Laser therapy + stretching exercises as in stretching group  Frequency: 2x/week,  Intensity: 3J/cm2  Duration: 60 min;10 sessions; 5 weeks | Type: Stretching exercises:  scalene, minor pectoralis, intercostals, the diaphragm, paraspinal, hamstring, glutei, triceps suralis, iliopsoas, adductors, internal rotators of the hip, trapezius, deltoid, elbow, fist and finger flexors, subscapular, major pectoralis, and coracobrachialis muscles  Frequency: 2x/week,  Intensity: 10 times  Duration: 60 min; 10 sessions; 5 weeks | VAS, FIQ pain, SF-36 pain  VAS:  Within Group (LSG):  -2.6(-5.09 to -0.10)  Within Group (SG):  -2.8(-4.77 to -0.82)  Between Group:  0(-2.27 to 2.27) | Reduces sensitivity of tender points  Improves bodily mechanics | h/o or suspicion of neoplasm |
| McCain et al. 1988 | Aerobic exercise:  Age: 38.5 (11.1)  Sex: 18 total  BMI: NR  Race: NR  Duration of symptoms: NR  Baseline Pain:70.1(15.8)  Flexibility exercise:  Age: 45.9 (8.2)  Sex: 20 women  BMI: NA  Race: NA  Duration of symptoms: NR  Baseline Pain: 56.3(19.2) | Type: Cardiovascular fitness: 10 min. warm-up, then bicycle ergometer  Frequency: 3x/week  Intensity: HR maintained in excess of 150 BPM for gradually increasing time periods  Duration: 60 min; 20 weeks | Type: Flexibility training: specifics not stated  Frequency: 3x/week  Intensity: HR < 115 beats per min  Duration: 20 weeks | VAS Pain, Total myalgic score  VAS:  Within Group (AG):  -23.2(-39.69 to -6.70)  Within Group (FG):  -8.9(-19.93 to 2.13)  Between Group:  0.5(-14.97 to 15.97) | Promotes Exercise induced hypoalgesia  Improves Mental status  Improves sleep | None |
| Mendonca et al. 2016 | tDCS and Aerobic exercise:  Age: 44.5 (14)  Sex: 1 male; 14 women  BMI: NR  Race: NR  Duration of symptoms (mo.): 140.6(72.2)  Baseline Pain:  7.2(1.75)  Aerobic exercise:  Age: 48 (11.8)  Sex: 15 women  BMI: NR  Race: NR  Duration of symptoms (mo.):  149.3(111.1)  Baseline Pain:  6.8(2.0)  tDCS:  Age: 49.9 (10.6)  Sex: 15 women  BMI: NR  Race: NR  Duration of symptoms: 125.6(100.2)  Baseline Pain: 7.2(1.27) | Type: tDCS + AE  AE: Each session, at the beginning and end of the treadmill exercise, the lower limbs were stretched  Frequency: 30 min  Intensity: Start at 60% HRmax, increased to 70% after second week  Duration: 4 weeks | Type: AE: Each session, at the beginning and end of the treadmill exercise, the lower limbs were stretched  Frequency: 30 min  Intensity: Start at 60% HRmax, increased to 70% after second week  Duration: 4 weeks  Frequency: NA  Intensity: NA  Duration: NA  Type: tDCS: No exercise | PPT, Visual Numeric Scale  #Mean values not reported; statistical significance was determined based on p values. | Promotes Exercise induced hypoalgesia  Long term potentiation  Motor cortex activation  Neurotransmitter release | medication for pain control for less than 2months, treated for depression for less than two months, undergoing some type of physical treatment for less than 2 months |
| Munguia-Izquierdo & Legaz-Arrese 2007 | Aquatic exercise group:  Age: 50 (7)  Sex: 29 women  BMI: 27 (5)  Race: 100% Caucasian  Duration of symptoms (yr.): 14(10)  Baseline Pain:  78.3(18.5)  Control group:  Age: 46 (8)  Sex: 24 women  BMI: 27 (4)  Race: 100% Caucasian  Duration of symptoms (yr.): 14(9)  Baseline Pain:  72.5(24.7) | Type: Aquatic exercise (combined) (chest high water: 32^°^ Celsius): 10 min. warm-up (slow walks and mobility exercises), 10-20 min. of strength exercises (using water and aquatic materials as resistance), 20-30 minutes of aerobic exercise, 10 min. cooldown (low-intensity and relaxation exercises)  Frequency: 3x/week  Intensity: 50-80% HRmax  Duration: 16 weeks | Type: Control: not stated  Frequency: NA  Intensity: NA  Duration: NA | VAS Pain, PPT  VAS:  Within Group (AG):  -11.4(-20.75 to -2.04)  Within Group (CG):  3.3(-9.75 to 16.35)  Between Group:  8.9(-0.68 to 18.48) | Promotes exercise induced hypoalgesia  Decreased sympathetic activity  Increased capillary density and decreased susceptibility to muscle trauma  Aquatic environment | history of morbid obesity, severe trauma, inflammatory rheumatic diseases, and severe psychiatric illness, who attended another physical or psychological therapy, a history of regular physical activity more strenuous than slow-paced walking a maximum of 2 times a week over 4 months |
| Mutlu et al. 2013 | Mixed exercise group:  Age: 45.63 (9.1)  Sex: 30 women  BMI: 28.83 (4.8)  Race: NR  Duration of symptoms: NR  Baseline Pain: NR  Combined exercise and TENS group:  Age: 43.30 (10.8)  Sex: 30 women  BMI: 28.95 (5.2)  Race: NR  Duration of symptoms: NR  Baseline Pain: NR | Type: Combined exercise: 5 min. warm up, 10 min. cycling, 20 min. stretching and strengthening, 5 min. cooldown.  Frequency: 3x/week  Intensity: 60-70% of max HR  Duration: 40 min; 12 weeks | Type: Exercise & TENS: Same as exercise in Group 1 + TENS  Frequency: 3x/week  Intensity: 60-70% HRmax  Duration: 12 weeks | Myalgic pain scores.  # Median and IQR reported; statistical significance is determined based on p values | Improves plasticity | Rheumatoid arthritis or other rheumatic diseases, psychiatric disorders. |
| Newcomb et al. 2011 | Both groups:  Age: overall age of participants: 44(12)  Sex: 11 women in the prescribed exercise group and 10 women in preferred exercise group  BMI: NR  Race: NR  Duration of symptoms:  Baseline Pain: NR | Frequency: 2 sessions; 2 weeks apart  Intensity: 60-75% age adjusted max HR  Duration:  Type: Prescribed, then preferred exercise: Prescribed - 20 min. cycling at 6--75 HRmax | Frequency: 2 sessions; 2 weeks apart  Intensity: self-selected intensity  Duration:  Type: Preferred, then prescribed exercise: Preferred - 20 min. at desired intensity | Pain thresholds,  Short Form Mc Gill pain questionnaire  #Mean values are not reported; statistical significance determined based on p values | Promote exercise induced hypoalgesia | major depressive disorder, current substance abuse, taking opioid or high-dosage antidepressant medications |
| Panton et al. 2009 | Resistance exercise group:  Age: 50 (7)  Sex: 10 women  BMI: 33.1 (7.9)  Race: NR  Duration of symptoms (yr.): 4(4)  Baseline Pain: NR  Resistance exercise and chiropractic group:  Age: 47 (12)  Sex: 11 women  BMI: 28.8 (5.8)  Race: NR  Duration of symptoms (yr.): 7(5)  Baseline Pain: NR | Frequency: 2/week  Intensity: one set of 8-12 reps 50% of one RM progressed to 100% of RM  Duration: 16 weeks  Type: 10 resistance exercise using 9 resistance machines; chest press, leg extension, leg curl, leg press, arm curl, seated dip, overhead press, seated row, abdominal crunch, and one body weight exercise for the lower back extension | Frequency: 2x/week, 8-12 reps per exercise  Intensity: Began a 50% 1RM and progressed to ~100%  Duration: 16 weeks  Type: Resistance Exercise + Chiropractic Treatment: Same exercises as group with additional chiropractic spinal adjustments. | Myalgic score  Within Group (RG):  -8(-13.35 to -2.64)  Within Group (R+CG):  -4.0(-8.53 to 0.53)  Between Group:  0(-4.53 to 4.53) | Slows down the cycle of deconditioning | uncontrolled diabetes, spinal trauma, spinal instability, long-term corticosteroid use, currently participating in an exercise program, and/or currently under the care of a chiropractic physician |
| Rodriguez-Mansilla et al. 2021 | Mixed exercise group:  Age: 52.24 (6.19) - average age of combined groups  Sex: 33 women  BMI: NA  Race: NA  Duration of symptoms:  Baseline Pain: 7.16(2.02)  Qigong exercise group:  Age: see above  Sex: 31 women  BMI: NR  Race: NR  Duration of symptoms: NR  Baseline Pain: 7.88(1.51)  Control group:  Age: see above  Sex: 29 women  BMI: NR  Race: NR  Duration of symptoms: NR  Baseline Pain: 7.34(1.61) | Type: Combined exercise program: 3-5 min warm-up (walking), active mobilization of shoulders, spine and hips, static balance exercises and stretches  Frequency: 2x/week, 45 min. Each mobilization exercise was completed in 6 reps of 10 seconds.  Intensity: NA  Duration: 4 weeks | Type: Qi Gong: Exercises combining mental concentration and abdominal breathing during performance of balance, flexibility and coordinated body movements.  Frequency: 2x/week, 45 min. Each figure repeated 6 times  Intensity: NA  Duration: 4 weeks  Control: Did not receive any treatment | VAS Pain  Within Group (CEG):  -1.09(-1.83 to -0.34)  Within Group (QG):  -1(-2.17 to 0.17)  Within Group (CG):  -0.03(-0.96 to 0.90)  Between Group:  (CE vs QG):  -0.63(-1.65 to 0.39)  Between Group:  (CE vs CG):  0.52(-0.33 to 1.37)  Between Group:  : (QG vs CG):  -1.15(-2.32 to 0.02) | Improves flexibility | Active exercise treatment previous to the study, regular physical exercise, or aerobic training, had previous knowledge of exercise for well-being. |
| Rooks et al. 2007 | Aerobic exercise group:  Age: 48 (11)  Sex: 35 women  BMI: 29 (6)  Race: White: 83% African American: 11 % Other: 6%  Duration of symptoms (yr.): 5(4)  Baseline Pain: 6.0(2.1)  Resistance exercise group:  Age: 50 (11)  Sex: 35 women  BMI: 30 (6)  Race: White: 91% African American: 3% Other: 6%  Duration of symptoms (yr.): 6(4)  Baseline Pain: 5.6(2.1)  Self-help course:  Age: 51 (12)  Sex: 51 women  BMI: 29 (7)  Race: White: 93% African American: 7% Other: 0%  Duration of symptoms (yr.): 6(5)  Baseline Pain:  6.01(2.1)  Resistance exercise and self-help course:  Age: 50 (11)  Sex: 50 women  BMI: 29 (6)  Race: White: 92% African American: 8% Other: 0%  Duration of symptoms (yr.): 6(6)  Baseline Pain: 6.6(2.1) | Type: Aerobic Exercise: 5 min. warm-up that progressed to 45 min. walking, flexibility exercises to end session.  Frequency: 3x/week  Intensity: NR  Duration: 16 weeks  Type: Strength Training: 5 min. warm-up that progressed to 20 min. walking, 25 min. strength training (chest press, seated row and leg press, bicep curls, triceps kickbacks and abdominal crunches), flexibility exercises to end session  Frequency: 3x/week  Intensity: 50% 1RM  Duration: 16 weeks | Type: Fibromyalgia Self-Help Course: teaches individuals with FM about the condition and self-management skills  Frequency: every 2weeks  Intensity: NA  Duration: 7 sessions  Type: Strength Training + Fibromyalgia Self-Help Course: Patients were enrolled in both the strength training and FSHC groups  Frequency: NR  Intensity: NR  Duration: NR | FIQ-Pain, SF-36 Bodily Pain  FIQ-Pain:  Within Group (AE):  -1.2 (-2.3 to 0.09)  Within Group (SG):  -0.4(-1.37 to 0.57)  Within Group (SHC):  -0.1(-1.27 to 1.07)  Within Group (SHC+SG):  -1.1 (-2.13 to -0.06)  Between Group:  AE vs SG:  0.4(-0.67 to 1.49)  AE vs SHC:  1.1(-0.11 to 2.31)  AE vs SHC+SG  0.1(-1.04 to 1.24)  SG vs SHC  0.7(-0.37 to 1.77)  SG vs SHC+SG  -0.3(-1.33 to 0.73)  SHC vs SHC + SG  -1(-2.07 to 0.07) | Improves Self-efficacy  Reduces Fear of pain | None |
| Sanudo et al. 2010 | Aerobic exercise group:  Age: 55.9 (1.6)  Sex: 18 women  BMI: 29.6 (1.1)  Race: NR  Duration of symptoms: NR  Baseline Pain: NR  Mixed exercise group:  Age: 55.9 (1.7)  Sex: 17 women  BMI: 27.6 (1.1)  Race: NR  Duration of symptoms: NR  Baseline Pain: NR  Control group:  Age: 58.6 (1.9)  Sex: 20 women  BMI: 29.7 (1.1)  Race: NR  Duration of symptoms: NR  Baseline Pain: NR | Type: Aerobic Exercise: 10 min. warm-up (slow walks, easy movements of progressive intensity), walks, easy movements, relaxation training)  15-20 min. of AE (continuous walking and jogging), 15 min. of interval training (aerobic dance and jogging), 5-10 min. of cool-down (slow  Frequency: 2/week  Intensity: AE: 60-65% HRmax Interval Training: 75-80% HRmax  Duration: 45 to 60 mins; 24 weeks | Type: Combined Exercise: AE and resistance training. 10 min. warm-up, 10-15 min. of AE, 15-20 min. of muscle strengthening (1 set of 10 reps for 8 different muscle group), and 10 min. of flexibility (1 set of 3 reps holding for 30 seconds for 9 different exercises). Muscles targeted: deltoids, biceps, neck, hip, back/chest/torso.  Frequency: 2/week  Intensity: AE: 65-70% HRmax  Duration: 45 to 60 min; 24 weeks  Control group:  Usual care medical treatment and normal ADLs | SF-36 Pain, FIQ Bodily Pain  #Mean values are not reported; statistical significance determined based on p values | Improved joint mobility | Inflammatory rheumatic diseases and severe psychiatric illness, receiving psychological or physical therapy |
| Sarmento et al. 2020 | Qigong group:  Age: 42.6 (10.7)  Sex: 10 women  BMI: NR  Race: NR  Duration of symptoms (yr.): 12.5(4.7)  Baseline Pain:  5.9(1.4)  Sham Qigong group:  Age: 56.1 (12.3)  Sex: 10 women  BMI: NR  Race: NR  Duration of symptoms (yr.):  12.6(5.0)  Baseline Pain: 7(1.5) | Type: Qigong: "six healing sounds": deep diaphragmatic breathing, mild body movements, and meditation along with uttering six healing sounds.  Frequency: one direct contact session per week 2 sessions per day at home  Intensity: NA  Duration: 25 minutes; 10 weeks | Type: Sham Qigong: Same body movements as the Qigong group, but healing sounds combined with diaphragmatic breathing and meditation were not taught.  Frequency: one direction contact session per week 2 sessions per day at home  Intensity: NA  Duration: 25 minutes; 10 weeks | VAS Pain, PPT, Short Form McGill Pain Questionnaire  VAS:  Within Group (QG):  -2.8(-4.26 to -1.33)  Within Group (SQ):  -0.2(-1.91 to 1.54)  Between Group:  -3.7(-5.49 to 1.90); Qigong group better | Immune regulation  HPA axis  Hormonal stress levels  Modulation of Autonomic activity  Down regulate Biomarkers | h/o regular opioid use, major depressive disorders, chronic inflammatory disorders, severe psychiatric illness |
| Sencan et al. 2004 | Aerobic exercise group:  Age: 35.40 (9.62)  Sex: 20 women  BMI: 24.14 (3.73)  Race: NR  Duration of symptoms(yr.): 4.68)4.18)  Baseline Pain: 6.85(1.23)  Paroxetine group:  Age: 32.65 (9.39)  Sex: 20 women  BMI: 24.25 (4.31)  Race: NR  Duration of symptoms(yr.): 6.53(5.63)  Baseline Pain:  6.62(1.42)  Placebo TENS:  Age: 35.55 (7.86)  Sex: 20 women  BMI: 24.60 (2.64)  Race: NR  Duration of symptoms(yr.): 5.10(4.68)  Baseline Pain: 7.70(1.72) | Type: Aerobic Exercise: 5 min. warm-up, 30 min. aerobic exercise, 5 min. cool-down.  Frequency: 3/week  Intensity: NR  Duration: 40 min; 6 weeks | Type: 20 mg/day paroxetine  Frequency: NA  Intensity: NA  Duration: 6 weeks  Type: placebo TENS  Frequency: 3x/week  Duration: 20 minutes | VAS; pressure algometry  VAS:  Within Group (AE):  -2.8(-3.49 to -2.10)  Within Group (PG):  -3.07 (-3.95 to -2.18)  Within Group (p TENS):  -1.5(-2.63 to -0.36)  Between Group:  (AE vs PG):  0.26(-0.76 to 1.28)  BG (AE vs p TENS)  1.09(-0.01 to 2.19)  BG (PG vs p TENS)  0.83(-0.45 to 2.11) | Positive effects on cardiovascular system  Release of endorphins | Tumors |
| Sevimli, 2015 | Age: 35.0 (8.8) Average age for all participants  Sex: 25 women in each of the three groups  BMI: NR  Race: NA  Duration of symptoms: NR  Baseline Pain:  Isometric exercise group: 68.2(11.8)  Aerobic exercise group: 70.0(12.9)  Aquatic exercise group: 71.5(13.1) | Type: Home-based isometric strength and stretching exercise:  Frequency: 7 days/week  Intensity: NA  Duration: 3 months | Type: Aerobic exercise program  Frequency: 2/week  Intensity: 60-80% HR max  Duration: 40-45 mins; 3 months  Type: Aquatic aerobic exercise program:  Frequency: 2/week  Intensity: 60-80% HR max  Duration: 40-45 mins; 3 months | VAS  Within Group (IG):  2.2(-4.71 to 9.11)  Within Group (AG):  -21.8(-28.07 to -15.52)  Within Group (AAG):  -23.5(-29.96 to -17.03)  Between Group:  IG vs AG:  -22.0(-28.31 to -16.08): AG better  IG vs AAG:  -22.4(-28.63 to -16.16)  AG vs AAG:  -0.2(-5.34 to 4.94) | Aquatic environment  Social effects | None/unclear criteria |
| Tomas-Carus et al. 2009 | Aquatic exercise group:  Age: 50.7 (10.6)  Sex: 15 women  BMI: 28.8 (4.5)  Race: NR  Duration of symptoms (yr.):  20.1(8)  Baseline Pain:  20.8(19.2)  Control group:  Age: 50.9 (6.7)  Sex: 15 women  BMI: 26.6 (3.5)  Race: NR  Duration of symptoms (yr.): 19.4(6.9)  Baseline Pain:  28.7(13.4) | Type: Aquatic Exercise  (33^°^ Celsius waist-high pool of water): 10 min warm up (slow walks and easy movements), 10 min. aerobic exercise, 20 min of overall mobility and strength (4 sets of 10 reps - knee flexion and extension), another 10 min. of aerobic exercise, 10 min cool down of low intensity exercises.  Frequency: 3x/week  Intensity: Aerobic Exercise: 60-65% HRmax  Duration: 1hr; 32 weeks | Control group:  Continued daily activities which did not include any psychological therapy or physical exercise similar to that in the program | SF-36 Bodily pain  Within Group (AG):  23(13.08 to 32.91)  Within Group (CG):  6.3(-8.71 to 21.31)  Between Group:  24.6(11.55 to 37.64); aquatic group better | Cardiorespiratory conditioning  - Aquatic environment  (warm water)  -Supervised group training | h/o sever trauma, frequent migraines, inflammatory rheumatic diseases, severe psychiatric illness, who attended to another psychological or physical therapy or who may have partaken in more than one exercise session of 30 min/week during a 2-week period in the last 5 years. |
| Tomas-Carus et al. 2007 | Aquatic exercise group:  Age: 51 (10)  Sex: 17 women  BMI: 27 (5)  Race: NR  Duration of symptoms (yr.): 24(9)  Baseline Pain: 21(19)  Control group:  Age: 51 (9)  Sex: 17 women  BMI: 27 (4)  Race: NR  Duration of symptoms (yr.): 19(8)  Baseline Pain: 23(19) | Type: Aquatic Exercise Group (33^°^ Celsius waist-high pool water): 10 min. warm up (slow walks and easy movements), 10 min. aerobic exercise (65-75% HRmax), 20 min. overall mobility and lower-limb strength exercises (4 sets of 10 of knee flexion and extension), another 10 min. of aerobic exercise, 10 min. cool down with low intensity exercise. 12 weeks between cessation of program and follow-up measurements.  Frequency: 3x/week  Intensity: 65-75% max HR 4 sets of 10 reps  Duration: One hour; 12-week period | Control group:  Type: continued their daily activities, which did not include any form of physical exercise similar to the intervention group | SF-36 bodily pain  Within Group (AG):  23(8.26 to 37.73)  Within Group (CG):  5(-8.62 to 18.62)  Between Group:  -16(-31.05 to 0.94) | Aquatic environment  -muscle relaxation (warm water)    -Endogenous opioids | history of severe trauma, frequent migraines, peripheral nerve entrapment, inflammatory rheumatic diseases, and severe psychiatric illness, who attended another psychological or physical therapy |
| Toprak Celenay et al. 2020 | Stabilization exercise group:  Age: 44(10) (median, IQR)  Sex: 21 women  BMI: 27.39(7.06)  Race: NR  Duration of symptoms: NR  Baseline Pain:7.86(1.81)  Stabilization exercise plus K taping:  Age: 38(24) (median, IQR)  Sex: 17 women  BMI: 24.8(4.63)  Race: NR  Duration of symptoms: NR  Baseline Pain:5.92(2.64) | Type: Spinal Stabilization Exercise: 10 min. warm-up, 10 min. SSE, and 10 min. cool-down. SSE aimed to maintain the neutral spine position and activate core muscles during exercise.  Frequency: 2x/week  Intensity: 10 s hold; 10 reps; 6 to 12 reps  Duration: 60 min; 6 weeks | Type: Spinal Stabilization Exercise + Kinesio Tape: Same exercise, but with the addition of Kinesiotaping before each session on the spine/scapulae  Frequency: 2x/week  Intensity:  Duration: 6 weeks | VAS Pain, FIQ Pain  # reported median and IQR, statistical significance is determined based on p values | Promotes neuromuscular control | malignancy, received any intervention including an exercise program or physical therapy in the previous 6 months |
| Valim, 2003 | Aerobic exercise group:  Age: Overall two group mean: 46.05(9.82)  Sex: 32 women  BMI: NA  Race: NA  Duration of symptoms: NR  Baseline Pain: 6.19(1.64)  Stretching exercise group:  Age: see above  Sex: 28 women  BMI: NR  Race: NR  Duration of symptoms: NR  Baseline Pain:  6(2.1) | Type: Aerobic Exercise: 5-10 min. warm-up of slow walking, aerobic exercise by walking, 5 min. of cool down (rhythmic movements).  Frequency: 3x/week  Intensity: Training heart rate was defined as the load beat immediately preceding the one in which the anaerobic threshold occurred  Duration: 45 min; 20 weeks | Type: Stretching Exercise: 17 exercises using both muscles and joints in a general way including face, cervical, trunk, and extremities - each maximum position sustained for 30 seconds.  Frequency: 3x/week  Intensity: Stretching exercises chosen to avoid heart rate increases.  Duration: 45 min | VAS score; SF-36  VAS:  Within Group (AE):  -2.77(-3.82 to -1.71)  Within Group (SG):  -1.4(-2.54 to -0.25)  Between Group:  -2.58(-3.78 to -1.37); Aerobic group better | Increasing peripheral levels of ß-endorphins influencing the monoamine-serotoninergic system, promoting a decrease in sympathetic activity, improving sleep, and improving psychological status | body mass index > 35; other rheumatic diseases |
| Vitorino et al. 2006 | Aquatic exercise group:  Age: 48.9 (9.2)  Sex: 24 women  BMI: NR  Race: NR  Duration of symptoms: NR  Baseline Pain: 27.4(15.5)  Physiotherapy group:  Age: 46.6 (8.4)  Sex: 23 women  BMI: NR  Race: NR  Duration of symptoms: NR  Baseline Pain: 12.0(12.8) | Type: Aquatic exercise: 5 min. warm-up, 6 min. stretching, 30 min. aerobic exercise, 13 min. relaxation, 6 min. stretching again  Frequency: 3x/week  Intensity: NR  Duration: 60 min' 3 weeks  Type: Aquatic exercise: 5 min. warm-up, 6 min. stretching, 30 min. aerobic exercise, 13 min. relaxation, 6 min. stretching again | Physiotherapy: 10 min. surface heating by infrared lamp, 5 min. stretching, 30 min. aerobic exercise, 10 min. relaxation, 5 min. stretching again.  Frequency: 3x/week  Intensity:  Duration: 3 weeks  Type: Conventional | SF-36 Bodily Pain  Within Group (AG):  37.3(28.26 to 46.33)  Within Group (PG):  46.9(39.46 to 54.33)  Between Group:  -5.8(-14.05 to 2.45) | Effects of Aquatic environment | None |
| Wigers, 1996 | Aerobic exercise group:  Age: 43 (9)  Sex: 2 men and 18 women  BMI: NR  Race: NR  Duration of symptoms (yr.):9(5)  Baseline Pain: 70(20)  Stress management group:  Age: 44 (12)  Sex: 2 men and 18 women  BMI: NR  Race: NR  Duration of symptoms (yr.): 11(10)  Duration of symptoms: 11(10)  Baseline Pain: 75(18)    Control group:  Age: 46 (9)  Sex: 1 male; 19 women  BMI: NR  Race: NR  Duration of symptoms (yr.): 11(9)  Baseline Pain: 67(14) | Type: Aerobic Exercise: 23 min. music session (warm up and two peaks of high intensity training), 15 min. of aerobic "games" (tag, ball games, etc.), cool down and thorough stretching.  Frequency: 3x/week  Intensity: 4 periods of high intensity training per session (60-70% HRmax, 18-20 min.)  Duration: 45 minutes/ 14 weeks | Type: Stress management treatment  cognitive-behavioral stress management package  Frequency: 2/week  Intensity:  Duration: 90 minutes  Control group:  Type: Treatment as usual; Aquatic therapy (n= 3), psychomotor treatment (n = l), tricyclic anti- depressants (low evening doses, n = 8), and, mostly when needed, low doses of: analgesics (n = 6), muscle relaxants (n = 3), hypnotics (n = 3), and tranquilizers (n = 2). | VAS Pain, Dolorimeter Score (kg)  VAS:  Within Group (AG):  -11(-25.44 to 3.44)  Within Group (SMG):  -18(-31.46 to -4.56)  Within Group (CG):  8(-4.88 to 20.88)  Between Group:  AG vs SMG:  -2(-16.23 to 12.23)  AG vs CG:  16(0.74 to 31.25); aerobic group better  SMG vs CG:  18(3.36 to 32.63); SMG better |  | Patients with aches and pains thought to be related to trauma (obvious or due to repetitive use). |
| Wong, 2018 | Tai chi group:  Age: 51 (2)  Sex: 17 women  BMI: 23.1 (0.5)  Race: Asian (Korean)  Duration of symptoms (yr.): 8(1)  Baseline pain: 7.5(0.4)  Control group:  Age: 51 (2)  Sex: 14 women  BMI: 22.2 (0.6)  Race: Asian (Korean)  Duration of symptoms: 9(1)  Baseline pain: 7.3(0.4) | Type: Tai Chi: 10 min. warm-up, 40 min. of Tai Chi practice and exercise, 5 min. cool down.  Frequency: 3/week  Intensity: 40-50% of the HR reserve  Duration: 55 minutes; 12 weeks | Control group:  Type: Did not participate in any supervised or unsupervised exercise protocol and were asked to maintain their regular lifestyle habits | VAS  Within Group (TG):  -2.2(-28 to -1.7)  Within Group (CG):  -0.3(-0.8 to 0.2)  Between Group:  1.7(1.41 to 1.98); TG better | Promotes autonomic modulation | severe psychiatric, medication changes in the previous year psychological or physical therapy, had a history of steady exercise or received exercise training in the last year |
